# Repurposing Drugs, Ongoing Vaccine, and New Therapeutic Development Initiatives Against COVID-19

**DOI:** 10.3389/fphar.2020.01258

**Published:** 2020-08-19

**Authors:** Rudra P. Saha, Ashish Ranjan Sharma, Manoj K. Singh, Saikat Samanta, Swarnav Bhakta, Snehasish Mandal, Manojit Bhattacharya, Sang-Soo Lee, Chiranjib Chakraborty

**Affiliations:** ^1^ Department of Biotechnology, School of Life Science & Biotechnology, Adamas University, Kolkata, India; ^2^ Institute for Skeletal Aging & Orthopedic Surgery, Hallym University-Chuncheon Sacred Heart Hospital, Chuncheon-si, South Korea

**Keywords:** coronavirus, vaccine development, SARS-CoV-2, repurposed drug, antiviral treatment, COVID-19

## Abstract

As the COVID-19 is still growing throughout the globe, a thorough investigation into the specific immunopathology of SARS-CoV-2, its interaction with the host immune system and pathogen evasion mechanism may provide a clear picture of how the pathogen can breach the host immune defenses in elderly patients and patients with comorbid conditions. Such studies will also reveal the underlying mechanism of how children and young patients can withstand the disease better. The study of the immune defense mechanisms and the prolonged immune memory from patients population with convalescent plasma may help in designing a suitable vaccine candidate not only for the current outbreak but also for similar outbreaks in the future. The vital drug candidates, which are being tested as potential vaccines or therapeutics against COVID-19, include live attenuated vaccine, inactivated or killed vaccine, subunit vaccine, antibodies, interferon treatment, repurposing existing drugs, and nucleic acid-based vaccines. Several organizations around the world have fast-tracked the development of a COVID-19 vaccine, and some drugs already went to phase III of clinical trials. Hence, here, we have tried to take a quick glimpse of the development stages of vaccines or therapeutic approaches to treat this deadly disease.

## Introduction

Severe Acute Respiratory Syndrome (SARS) caused by SARS Coronavirus (SARS‐CoV) initially occurred in China (November 2002) and then quickly spread to 29 countries, resulted in 8,096 cases with 774 fatalities (mortality rate 9.6%). SARS was officially contained in July 2003, about eight months since its first outbreak (WHO, 2003; Peiris et al., 2004). MERS (Middle East Respiratory Syndrome) caused by MERS‐CoV (MERS Coronavirus) has resulted in a similar outbreak by spreading into 26 countries with 2519 infected cases and 866 deaths (mortality rate 34.4%) after its first report on June 2012 in Saudi Arabia (Assiri et al., 2013; World Health Organization, 2019). The current outbreak of COVID-19 (Coronavirus Disease 2019) caused by SARS-CoV-2, which was first reported in the Wuhan (China) on December 2019 (Hubei province), now gradually spilled over 213 countries and territories resulted in over 16.3 million infected cases with and more than 650,000 deaths (4% mortality rate) as of July 26, 2020 (Wang et al., 2020a). On January 30, 2020, WHO announced the current coronavirus outbreak as a world health emergency, and on March 11, 2020, reclassified it as a pandemic (World Health Organization, 2005; Chakraborty et al., 2020c; WHO, 2020). The virus was initially named Novel Coronavirus 2019 (2019-nCoV), and later it was changed to SARS-CoV-2 (Gorbalenya, 2020). The WHO entitled the disease as COVID-19 on February 11, 2020 (World Health Organization, 2020). The SARS-CoV-2 was found to be infectious as it spreads *via* respiratory droplets and aerosols when an infected individual comes in contact with a healthy person (Chan et al., 2020b; Liu Y. et al., 2020). The virus incubates for about 2–14 days within humans and subsequently resulted in various mild to severe symptoms like fever, dry cough, dyspnea, severe respiratory issues, pneumonia, etc (Chakraborty et al., 2020a; Chan et al., 2020b; Huang et al., 2020; Lauer et al., 2020; Zu et al., 2020).

Coronaviruses are ssRNA (positive-sense) virus and enveloped with a diameter of 80–120 nm (Sipulwa et al., 2016). This virus (SARS-CoV-2) under the beta-coronavirus genus of the *Coronaviridae* family comprises four genera—α-CoV, β-CoV, *γ*-CoV, and δ-CoV (Chan et al., 2013). Like SARS-CoV-2, MERS-CoV and SARS-CoV are also belonged to the genus β-CoV (Chan et al., 2013). Further, four HCoVs that cause mild symptoms, i.e., common cold, belong to the genera α-CoV (HCoV-NL63 and HCoV-229E) and β-CoV (HCoV-OC43 and HCoV-HKU) (Rabi et al., 2020). The size of the SARS-CoV-2 genome was found to be about 29.9 kb (GenBank Accession Number: MN908947.3) (Wu F. et al., 2020). Preliminary studies suggested that the genome of SARS‐CoV‐2 is closer to SARS‐CoV than MERS-CoV depending on the percentage similarity, although the highest genome similarity was found with the RaTG13 virus found in bats which indicated a plausible origin of SARS-CoV-2 (bat) (Chakraborty et al., 2020b; Lu et al., 2020; Zhou et al., 2020). Both SARS-CoV-2 and SARS-CoV uses the human ACE2 as a receptor for their entrance in the cell (Ge et al., 2013; Wan et al., 2020; Wrapp et al., 2020).

The cell membrane attached ACE2 converts the vasoconstrictor peptide angiotensin II to angiotensin 1–7 (vasodilator peptide), and it protects the heart and blood vessels (Jiang et al., 2014). ACE2 is found in the heart, lung, kidney, endothelium, etc. and known to reduce the adverse effects of other RAS (Renin-Angiotensin System) components by reducing the concentration of angiotensin II and increasing the concentration of angiotensin 1–7 and regulates the blood pressure in the body. ACE2 also found to express in intestinal epithelial cells where it helps to absorb nutrients from the food particles and was predicted as one of the entry sites that may have been used initially by SARS-CoV-2 upon the consumption of contaminated food from Wuhan seafood market (Hashimoto et al., 2012; Zhang et al., 2020a). Similarly, ACE2 is also found to express on the mucosa of the oral cavity and the epithelial cell of the tongue, making these other entry routes for SARS-CoV-2 (Xu et al., 2020). Interestingly, a small subset of type II alveolar cells (AT2) was found to express the ACE2 receptor and several other genes that positively regulate viral reproduction and transmission, making the lung more susceptible to the virus. The ACE2 expressing cells in the lung triggers an immune response, which may overreact to damage the lung cells by filling up the air sacs with fluid instead of gas, causing pneumonia. Patients with a severely damaged lung can develop acute respiratory distress syndrome (ARDS), where breathing becomes difficult (Li et al., 2020). As ACE2 expresses in an array of organs, SARS-CoV-2 can attack several organs, which results in multi-organ failure often observed in patients who died of COVID-19 (Wang T. et al., 2020). Patients with chronic cardiovascular diseases often take drugs that block the angiotensin receptor or inhibit the angiotensin-converting enzyme, which in turn increases the expression of ACE2 receptors in cells. Therefore, COVID-19 patients who regularly take these medications might have an increased hazard of SARS-CoV-2 infection (Diaz, 2020).

**Figure 1:**
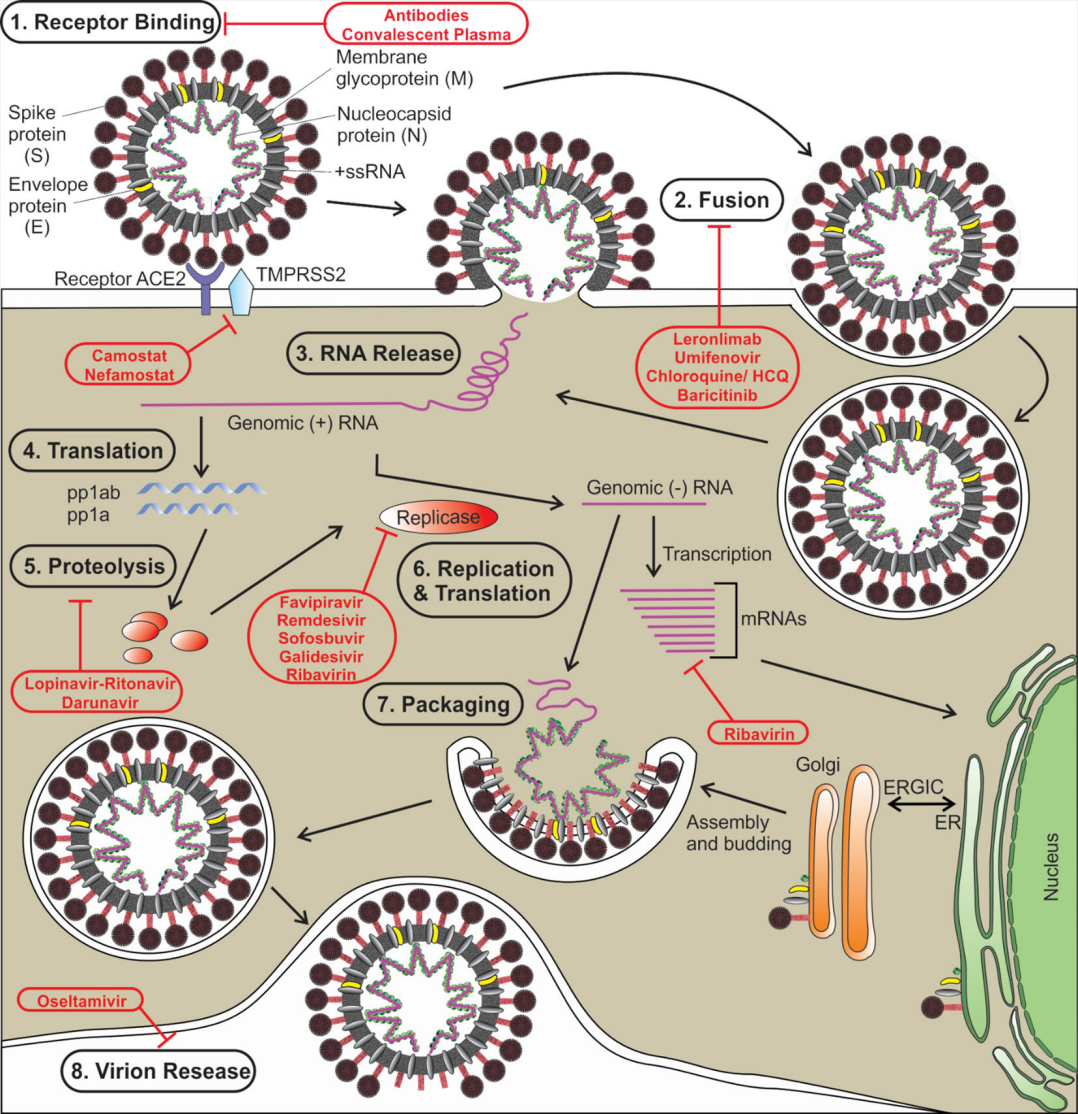
The life cycle of SARS-CoV-2 is shown. Various steps in the life cycle are mentioned—receptor binding of the virus, fusion with the host membrane, viral RNA release, translation of viral RNA, proteolysis of the proproteins, replication and translation, packaging of viral particles, and virion release. Possible targets of various antiviral drugs that are being repurposed/investigated for COVID-19 are indicated. S, spike protein; E, envelope protein; M, membrane protein; N, nucleocapsid protein; HCQ, hydroxychloroquine, ER, endoplasmic reticulum; ERGIC, ER-Golgi intermediate compartment.

Like other coronaviruses, SARS‐CoV‐2 also consists of two types of protein structural proteins and non-structural. Structural proteins comprise of E (envelope) protein, S (spike) protein, M (membrane) protein, and N (nucleocapsid) protein (Wu A. et al., 2020). The spike protein (S) of SARS‐CoV‐2 is a trimeric class I type of fusion protein that helps the virus to enter host cells (Bosch et al., 2003; Walls et al., 2020). The spike protein has two subunits, S1 (required for receptor recognition) and S2 (required for membrane fusion). The C-terminal RBD (receptor-binding domain) of the first subunit (S1 subunit) of spike protein directly interacts with the ACE2 receptor (Yuan et al., 2020). Upon the fusion of the S protein, which exists in a metastable prefusion state, with the ACE2 receptor, the S protein undergoes a conformational rearrangement. The binding to the ACE2 destabilizes the prefusion trimer, which results in the discharge of the S1 subunit. This allows the transition of the S2 subunit of S protein to a steady postfusion state (de Wilde et al., 2017). A cellular serine protease TMPRSS2 plays a pivotal role in this S protein priming (Hoffmann et al., 2020; Wrapp et al., 2020). The host cell-mediated S protein priming is an essential step for the virus to move into the host cells (Hoffmann et al., 2018). Once inside of the host cell, SARS-CoV-2 follows the typical life cycle of a positive-sense RNA virus as was found with MERS-CoV and SARS-CoV (**Figure 1**) (Fehr and Perlman, 2015).

According to some mathematical models, the transmission of the disease may quickly rebound if we relax measures like lockdown and social distancing (Yamey et al., 2020). In the absence of effective prophylactic treatment, such eruptions may leave the health system overburdened. The absence of a potential drug or vaccine against SARS-CoV-2 has already resulted in a pandemic situation (Wang D. et al., 2020). The designing and development of the COVID-19 vaccine that can be used globally is, therefore, the utmost priority for ending the current pandemic (Prompetchara et al., 2020). It was observed that both SARS‐CoV-2 and SARS‐CoV use the same mechanism to enter target cells has vital significance for our understanding of the SARS‐CoV‐2 pathogenesis and transmissibility. To fight this pandemic, various government and private organizations have sped up their development of vaccines and treatment procedures. In this review article, we have discussed the testing of various existing drugs that are now being repurposed and targets against which various vaccine developments are going on for COVID-19.

## Host Immune Response to Viral Infections

Upon viral infection, the host cell initially activates the innate immune response *via* PRRs (pattern-recognition receptors) that recognizes viral particles (Takeuchi and Akira, 2009). Host cells release a group of signaling proteins called Interferons (IFNs) that play a significant role in host antiviral defense. INFs belong to a group of peptides and proteins called cytokines responsible for transferring signals by binding to the receptors on the surface of appropriate immune cells for triggering host immune response against pathogens. INFs are triggered by the activation of host PRRs. Four types of PRRs are known—TLRs, RLRs, NLRs, and CLRs although during viral infection mainly three types of PRRs are activated—RLRs, TLRs, and NLRs (González-Navajas et al., 2012; Fehr and Perlman, 2015; Nan et al., 2018; Zhang et al., 2020b). PRRs recognize several viral components including DNA, ssRNA, dsRNA, RNA with 5′-triphosphate ends, and proteins. Detection of viral particles by PRRs activates signaling pathways that release type I INFs, different types of cytokines such as proinflammatory cytokines (primarily IL-1, IL-6, TNF-α), chemokines, and co-stimulatory molecules like CD40, CD80, and CD86 that results in inflammation and subsequent engagement of innate and acquired immune cells to eliminate viral infection (González-Navajas et al., 2012; Khan et al., 2012; Nan et al., 2018).

Three types of INFs have been characterized till now—type I IFNs (mainly IFN-α/β), type II IFNs (IFN-γ), and type III (INF-λ) (Stanifer et al., 2019). INFs-α/β is secreted by all viral-infected cells including pDCs (plasmacytoid dendritic cells) which is a vital cell type for INF-α secretion during viral infection. IFN-γ, secreted by NK (natural killer) cells and immune cell-like T cells, plays a vital role in host adaptive and innate immunity. It also regulates the expression of several genes that are affected by type I IFNs. INF-λ is mainly secreted by epithelial cells in response to the viral infection at mucosal sites (Zanoni et al., 2017). INFs protect host cells by activating signaling pathways, mainly the JAK/STAT pathway (Schindler et al., 1992; Darnell et al., 1994), which subsequently trigger the expression of ISGs (IFN-stimulated genes) that controls the viral infection (Katze et al., 2002). The activated STAT proteins (STAT1, STAT2, and STAT3) in response to INF stimulation are vital for transferring signals that subsequently activate ISGs (Levy and Darnell, 2002; Tsai et al., 2019). Type I INFs induced during innate immune response also upregulate several ISGs whose expression restricts viral replication (Kane et al., 2016).

Activation of the innate immune cells is critical for setting up adaptive immune responses during the re-infection by the same virus. Activation of adaptive immunity takes a few days to weeks to become established. APCs (antigen-presenting cells; e.g., dendritic cells, B cells and macrophages), that live at the site of viral infection, binds to viral particles (antigens) and present them on major histocompatibility complex (MHC) class II to be recognized by the T cell receptor on CD4^+^ T cells in presence of co-stimulatory molecules (Rosendahl Huber et al., 2014). The activated CD4^+^ T cells release a wide range of cytokines and chemokines that helps to differentiate CD4^+^ T cells into several cell subtypes, mainly T helper cells (such as Th1, Th2, Tfh, etc.) as well as regulatory T cells (Treg). Th1 and Th2 cells release several cytokines (Th1-INF-*γ*; Th2- IL-4, IL-13, IL-5, etc.) to trigger B cell differentiation and activate macrophages (Rosendahl Huber et al., 2014). T follicular helper cells (Tfhs) also helps to activate B cells to produce specific antibodies against foreign pathogens (Crotty, 2014). Treg cells do several regulatory functions, especially controlling immunopathology (Crotty, 2014). Activated CD4^+^ T cells by its interaction with the APCs through CD40-CD40L upregulate expression of CD80/CD86 markers on APCs which interacts with the CD28 on the CD8^+^ T cells. The APCs presents viral particles on the MHC class I molecules that bind to the TCRs on the CD8^+^ T cells through the CD80/CD86-CD28 interactions and activate CD8^+^ T cells. The activated cells proliferate and differentiate into CTLs (cytotoxic T lymphocytes) which releases cytotoxic molecules, and activates the production of cytokines (e.g., TNF-α, IL-2, IFN-*γ*, etc.) that promotes apoptosis of virally infected cells (Crotty, 2014).

Both innate and adaptive immunity (humoral and cell-mediated) are equally important to control viral infections. Innate immunity mounts host defenses to control viral infection at the early phases by releasing proinflammatory molecules and also activates adaptive immunity by upregulating co-stimulatory molecules. In adaptive immunity, B cells (humoral immunity) and T cells (cell-mediated immunity) are activated that prevent further viral infections. Immunoglobulins (IgG, IgM, and IgA) produced by activated B lymphocytes bind to viruses to block viral spread and also eliminate virus-infected cells *via* ADCC (antibody-dependent cytotoxic cells) or complement-mediated pathways. CTLs differentiated from activated CD8^+^ T cells kill the virus-infected cells by releasing cytotoxic cytokines that trigger apoptosis of the target cells. Some of these immune cells (T cells and B cells) are converted into memory cells that prevent further infections and provide long-term immunity (Klimpel, 1996).

SARS-CoV and other coronaviruses are sensitive to IFN-α/β. Some of these viruses are also very pathogenic. It might be attributed to their ability to modulate an effective host immune response. The nucleocapsid protein of SARS-CoV can evade host interferon responses (Spiegel et al., 2005; Kopecky-Bromberg et al., 2007; Lu et al., 2011). It was reported that EV71 (Liu et al., 2014) and Ebola virus infections can downregulate the JAK-STAT pathway mediated by type-I IFNs, and promote viral replication and proliferation within the host (Okumura et al., 2010). Several antibodies, for example, MCA1, CSCC5, CDC-C2, CDC-A10, CDC-A2, MERS-GD27, etc., isolated from recovered MERS-CoV-infected patients have been found useful in controlling the disease (Chen et al., 2017; Niu et al., 2018a; Niu et al., 2018b). Recognition mechanisms involving the surface proteins of virus and the receptors of host are vital for an understanding of the cross-species transmission and host tropism to establish animal models for effective vaccine development (Ahn et al., 2020).

Some COVID-19 patients with severe symptoms experience a sudden surge of cytokines in the body, released by the immune cells in response to the viral infection, commonly referred to as ‘cytokine storm’ (Huang et al., 2020). The excessive release of the cytokines or cytokine release syndrome (CRS) is a major determinant in inducing ARDS in COVID-19 patients. The excessive secretion of proinflammatory cytokines (e.g., IL-6, IL-1, TNF-α, etc.) with the help of the innate immune system within the body leads to several lung complications like pneumonitis and ARDS which can cause multi-organ failure and death (Nicholls et al., 2003; Mahallawi et al., 2018; Ragab et al., 2020). Among various proinflammatory cytokines, IL-6 plays a major role in inducing ARDS as an increase in the concentration of IL-6 in the plasma was found to be linked with ARDS in COVID-19 patients (Ragab et al., 2020). Association of IL-6 to mIL-6R (membrane-bound IL-6 receptor) and gp130 activates the JAK-STAT3 pathway which contributes toward CRS. Besides, at high concentrations, IL-6 binds to sIL-6R (soluble form of IL-6 receptor) and gp130, and activates JAK-STAT3 pathway in cells that do not express mIL-6R which again induces cytokine storm by releasing several cytokines and chemokines (e.g., VEGF, IL-6, MCP-1/CCL2, IL-8, etc.), and by reducing E-cadherin production that leads to ARDS (Magro, 2020; Ragab et al., 2020). Therefore, preventing the occurrence of cytokine storm by drugs that inhibits the release of cytokines may help in alleviating severe COVID-19 symptoms.

## Viral and Host Protein Targets

### Vaccines

SARS-CoV-2 expresses four structural proteins, N (nucleocapsid), E (envelope), S (spike) protein, and M (membrane) similar to SARS-CoV. These proteins are potential antigens to induce nAbs (neutralizing antibodies) and provide protective functions (Bhattacharya et al., 2020a; Chan et al., 2020a; Shang et al., 2020). So, the finding of a protein that has the dominant neutralizing epitopes should be the first step of the investigation. Before this identification, the inactivated virus can also be used as a first-generation vaccine because it is probably easier to generate than the whole-killed virus particles. Whole-cell killed or live-attenuated vaccines represent all the antigens present in a pathogen like proteins, nucleic acids, polysaccharides, lipids, and some other components capable of inducing a potent immune response (Sharma et al., 2011). Several studies have shown that SARS-CoV inactivated through an agent such as formaldehyde, β-propiolactone and UV light can also instigate virus-neutralizing antibodies in immunized animals (He et al., 2004; Xiong et al., 2004; Jiang et al., 2005; Qu et al., 2005; Te-hui et al., 2005). So in principle, inactivated SARS-CoV-2–based vaccines can also be used. However, upon identification of the neutralizing epitopes, the vaccines that are made based on fragments containing neutralizing epitopes should be used, as they are safer and more effective than the inactivated virus vaccine. Several organizations are using viral deoptimization techniques to synthesize more effective vaccines such as live-attenuated vaccines (Zhang J. et al., 2020). Though, attenuated vaccine mimics the natural course of infection to stimulate the toll-like receptors e.g. (TLR-3, TLR-4, TLR-7, TLR-8, and TLR-9) and provide long-term immunity, ensuring low or no pathogenicity is always a major concern (Chakraborty et al., 2020d). Also, killed vaccines show difficulty in maintaining consistency in quality (Chen W. H. et al., 2020).

Most of the subunit vaccines against coronaviruses depend on mounting immune responses against the spike protein by preventing its binding to the host ACE2 receptor (Jiang et al., 2012). One way to block access to the entry receptor, i.e., human ACE2 receptor is to use the spike protein RBD (receptor-binding domain) of SARS-CoV-2 that has been shown to attach to the ACE2 receptor (Lan et al., 2020). Spike protein’s RBD from SARS-CoV has been shown to block the virus from accessing the ACE2 receptor in cell culture (Wong et al., 2004). Besides, the RBDs of spike proteins in both SARS-CoV-2 and SARS-CoV were found to interact similarly with the ACE2 receptor (Lan et al., 2020). Other researchers have proposed that the RBDs on the spike proteins of other coronaviruses like MHV (mouse hepatitis virus), TGEV (transmissible gastroenteritis virus), HCoV-229E, SARS-CoV, etc. contain key antigenic determinants that can induce production of neutralizing antibodies (Godet et al., 1994; Kubo et al., 1994; Bonavia et al., 2003; He et al., 2004). As spike proteins of coronaviruses are the most important antigenic determinants known to trigger neutralizing antibodies, spike proteins can be used as antigens for developing vaccines (Saif, 1993; Schmidt et al., 2006; Bhattacharya et al., 2020a; Bhattacharya et al., 2020b). Spike protein RBD sequences are relatively conserved. So, this may possible to find the neutralizing epitopes present into the SARS-CoV-2 spike protein for designing and developing of effective, safe vaccine against this virus. How spike protein RBD can activate extremely effective neutralizing antibodies against this virus has been elucidated by the mAbs (monoclonal antibodies) which was isolated from the inactivated virus-immunized human and mice antibody libraries (Sui et al., 2004; He et al., 2005). Thus, the RBD of this virus S protein is not only a functionally important domain for receptor binding of this virus but also a significant neutralization determinant element of SARS-CoV-2. So, the proteins that contain the RBD region or vectors encoding the spike protein RBD can be utilized for developing a highly effective vaccine candidate (**Table 1**). Therefore, the RBD alone could block access to ACE2 for SARS-CoV-2. Alternatively, single-domain antibodies (sdAbs) or nanobodies based on the RBD can also block the ACE2 receptor effectively (Arbabi-Ghahroudi, 2017). Researchers are developing virus-like nanoparticles based on the expression of recombinant spike protein, which can act as a potent immunogen. Others have developed subunit vaccines consisting of the RBD from SARS-CoV S protein (Chen W. H. et al., 2020). However, certain limitations of subunit vaccines exist, for example, the requirement of multiple booster shots and suitable adjuvants (Shang et al., 2020).

**Table 1:** Ongoing vaccine development initiatives against COVID-19 by different organizations that are at different phases of clinical and preclinical trials (updated on July 25, 2020).

No.	Clinical/preclinical stage	Vaccine name/type	Remark	Organization/Company
1	Phase IV	Oral polio vaccine	mixture of live attenuated poliovirus strains	Bandim Health Project, Denmark
2	Phase IV	BCG vaccine	live attenuated bacteria	Merck & Co. Inc., USA
3	Phase III	mRNA-1273	LNP-encapsulated mRNA	Moderna Therapeutics Inc., USA
4	Phase III	Viral vaccine	Inactivated vaccine	Sinopharm, China; Wuhan Institute of Biological Products, China
5	Phase III	Coronavac	Inactivated + alum	Sinovac Biotech Ltd., China; Dynavax Technologies, USA; Instituto Butantan, Brazil; PT Bio Farma, Indonesia
6	Phase II	Ad5-nCoV	nonreplicating viral vector (Adenovirus Type 5 Vector)	Cansino Biologics Inc., China; The Beijing Institute of Biotechnology of the Academy of Military Medical Sciences, China
7	Phase I/II	AV-COVID-19	autologous dendritic cells loaded with antigens from SARS-CoV-2	Aivita Biomedical Inc., USA
8	Phase I/II	AG0301-COVID19	DNA plasmid vaccine	Anges Inc., Japan; Osaka University, Japan; Takara Bio Inc., USA; Japan Agency for Medical Research and Development, Japan
9	Phase I/II	AZD-1222 (formerly ChAdOx1 nCoV-19)	nonreplicating viral vector-based	Astrazeneca, UK; The Jenner Institute, UK; University of Oxford, UK; Oxford Biomedicaplc, UK; Vaccines Manufacturing and Innovation Centre, UK; Pall Life Sciences, USA; Cobra Biologics, UK; Halix BV, Netherlands; Emergent Biosolutions Inc., USA; Catalent Inc., USA
10	Phase I/II	Covaxin	inactivated whole-virion vaccine	Bharat Biotech International Ltd., India
11	Phase I/II	BNT-162	RNA vaccine; 3 LNP-mRNAs	Biontech AG, Germany; Shanghai Fosun Pharmaceutical Co. Ltd., China; Pfizer Inc., USA
12	Phase I/II	SARS-CoV-2 vaccine	Inactivated vaccine	Chinese Academy of Medical Science, China; West China Second University Hospital, China; Yunnan Center for Disease Control and Prevention, China
13	Phase I/II	Gam-COVID-Vac	nonreplicating viral vector (Adeno-based)	Gamaleya Research Institute of Epidemiology and Microbiology, Russia; Health Ministry of the Russian Federation, Russia; Acellena Contract Drug Research & Development
14	Phase I/II	GX-19	DNA Vaccine	Genexine Inc., South Korea; PT Kalbe FarmaTbk, Indonesia
15	Phase I/II	V-SARS	made from heat-inactivated plasma from donors with COVID-19	Immunitor LLC, Canada
16	Phase I/II	COVAC1	RNA vaccine (saRNA)	Imperial College, UK
17	Phase I/II	INO-4800	DNA plasmid vaccine	Inovio Pharmaceuticals Inc., USA; Beijing Advaccine Biotechnology Co. Ltd., China; Geneone Life Science Inc., South Korea; Ology Bioservices Inc., USA; International Vaccine Institute, South Korea
18	Phase I/II	KBP-COVID-19 vaccine	protein subunit vaccine; RBD-based	Kentucky Bioprocessing (KBP), USA; U.S. biotech subsidiary of British American Tobacco (BAT)
19	Phase I/II	Allostim vaccine	bioengineered cells to provide protection from different viral infections	Mirror Biologics Inc., USA; Immunovative Therapies Ltd., Israel; Hadassah-Hebrew University Medical Center, Israel
20	Phase I/II	NVX-CoV2373	protein subunit vaccine; full length recombinant SARS-CoV-2 glycoprotein nanoparticle vaccine adjuvanted with Matrix M	Novavax Inc., USA
21	Phase I/II	Adenoviral vector vaccine	nonreplicating viral vector; replication defective Simian Adenovirus (GRAd) encoding SARS-CoV-2 S	Reithera Srl, Italy; Leukocare AG, Germany; Univercells SA, Belgium
22	Phase I/II	LV-SMENP-DC	lentiviral vector system that express viral proteins and immune modulatory genes	Shenzhen Geno-immune, China
23	Phase I/II	BBIBP-CorV	Inactivated vaccine	Sinopharm, China; Beijing Institute of Biological Products Co. Ltd., China; Henan Provincial Center for Disease Control and Prevention, China
24	Phase I/II	ZyCov-D	plasmid DNA vaccine	Zydus Cadila, India
25	Phase I	LUNAR-COV19 (ARCT-021)	RNA vaccine (mRNA)	Arcturus Therapeutics Holdings Inc., USA; Duke-NUS Medical School, Singapore
26	Phase I	SCB-2019	protein subunit vaccine; native-like trimeric subunit spike protein vaccine	Clover Biopharmaceuticals Inc., China; Glaxosmithkline plc., UK; Dynavax Technologies Corp., USA
27	Phase I	DNA vaccine	DNA with electroporation	Cobra Biologics Ltd., UK; Karolinska Institutet, Sweden
28	Phase I	CVnCoV	RNA vaccine (mRNA)	Curevac AG, Germany
29	Phase I	RUTI vaccine	replicating viral vector; attenuated influenza expressing an antigenic portion of the spike protein	Fundacio Institut Germans Trias i Pujol, Spain
30	Phase I	COVAX-19	spike protein-based vaccine	Genecure Biotechnologies, USA; Vaxine, Australia; Medytox, South Korea
31	Phase I	DPX-COVID-19	protein subunit vaccine; peptide antigens formulated in LNP	IMV Inc., Canada; University Laval, Canada
32	Phase I	IPT-001	peptide-based vaccine	Intellistem Technologies Inc., Canada
33	Phase I	Virus-like particle vaccine; CoVLP	plant-derived VLP; CpG 1018 and pandemic adjuvant	Medicago Inc., Canada; Glaxosmithkline plc., UK
34	Phase I	Adjuvanted recombinant subunit vaccine	S protein (baculovirus production)	Sanofi SA, France; Glaxosmithkline plc., UK
35	Phase I	aAPC vaccine	lentiviral vector system to express SARS-CoV-2 minigenes engineered based on multiple viral genes	Shenzhen Geno-immune Medical Institute, China
36	Phase I	bacTRL-Spike	DNA vaccine	Symvivo Corp., Canada
37	Preclinical	mRNA vaccine	needle-free injection system to deliver mRNA	Abnova Corp., Taiwan; Pharmajet Inc., USA
38	Preclinical	SARS-CoV-2 vaccine	saponin-based adjuvant TQL-1055 with SARS-CoV-2 antigen	Adjuvance Technologies Inc., USA; National Institutes of Health, USA
39	Preclinical	MAPS vaccine	polysaccharide and the protein-based multiple antigen presenting system	Affinivax Inc., USA
40	Preclinical	Vaccine	protein subunit vaccine based on Spike protein	AJ Vaccines, Denmark
41	Preclinical	COVID-19 vaccine	triple antigen VLP vaccine	Akers Biosciences Inc., USA; Premas Biotech Pvt Ltd., India
42	Preclinical	Chimigen vaccine	recombinant protein vaccine	Akshaya Bio Inc., Canada; Cytovance Biologics, USA; Shenzhen Hepalink Pharmaceutical Group Co. Ltd., China
43	Preclinical	AdCOVID	nonreplicating viral vector; adenovirus-based NasoVAX expressing SARS-CoV-2 spike protein	Altimmune Inc., USA; University of Alabama at Birmingham, USA
44	Preclinical	COVID-19 vaccine	VLP vaccine	Artes Biotechnology GmbH, Germany
45	Preclinical	Recombinant coronavirus vaccine	spike protein-based	Autonomous University of Mexico (UNAM), Mexico
46	Preclinical	COVID-19 vaccine	spike protein-based	Autonomous University of Queretaro (UAQ), Mexico
47	Preclinical	Vaccine	protein subunit vaccine; based on peptides derived from spike protein	Axon Neuroscience SE, Cyprus
48	Preclinical	Vaccine	protein subunit vaccine; S1 or RBD of spike protein	Baylor College of Medicine, USA; New York Blood Center, USA; Fudan University, China
49	Preclinical	Vaccine	universal dendritic cell vaccine	Betta Pharmaceuticals Co. Ltd., China; Beijing Dingcheng Taiyuan Biotechnology, China
50	Preclinical	Vaccine	DNA vaccine	Bionet Asia, Thailand
51	Preclinical	SARS-CoV-2 vaccine	recombinant subunit vaccine	Chongqing Zhifei Biological Products Co. Ltd., China; Institute of Microbiology, Chinese Academy of Sciences, China
52	Preclinical	Vaccine	protein-based vaccine	Coalition for Epidemic Preparedness, Norway; Dynavax Technologies Corp., USA
53	Preclinical	CDX-005	live attenuated virus; codon deoptimized live attenuated vaccine	Codagenix Inc., USA; Serum Institute of India Ltd., India
54	Preclinical	Vaccine	multitope peptide-based vaccine (MPV)	Covaxx, a unit of United Biomedical Inc., USA
55	Preclinical	Vaccine	RNA vaccine; LNP-encapsulated mRNA	Daiichi Sankyo, Japan; University of Tokyo, Japan
56	Preclinical	Vaccine	develped on hyper-productive C1 gene-expression platform	Dyadic International Inc., USA; The Israel Institute for Biological Research, Israel
57	Preclinical	Vaccine	protein-based vaccine	Eijkman Institute for Molecular Biology, Indonesia; PT Bio Farma, Indonesia
58	Preclinical	EXG-5003	self-replicating RNA (srRNA) vaccine	Elixirgen Therapeutics Inc., USA
59	Preclinical	Covigenix	Fusogenix DNA vaccine	Entos Pharmaceuticals, Canada
60	Preclinical	Vaccine	vaccine contain virions, viral proteins at different stages of viral replication	Epitopoietic Research Corp., Belgium
61	Preclinical	EPV-CoV19	protein subunit vaccine; spike protein	Epivax Inc., USA; University of Georgia, USA
62	Preclinical	mRNA vaccine	RNA vaccine; mRNA in an intranasal delivery system	Etherna Immunotherapies NV, Belgium
63	Preclinical	Vaccine (protein subunit; virus-like particle)	drosophila S2 insect cell expression system VLPs	ExpreS2ion Biotechnologies ApS, Denmark; Adaptvac ApS, Denmark; AGC Biologics, Denmark; Bavarian Nordic A/S, Denmark
64	Preclinical	Flowvax	protein subunit vaccine; peptide	Flow Pharma Inc., USA; University of Texas Medical Branch at Galveston, USA
65	Preclinical	Coroflu	replicating viral vector; M2-deficient single replication (M2SR) influenza vector	Flugen Inc., USA; Bharat Biotech International Ltd., India; University of Wisconsin-Madison, USA
66	Preclinical	Vaccine	RNA vaccine; LNP-encapsulated mRNA cocktail encoding VLP	Fudan University, China; Shanghai Jiao Tong University, China; RNACure Biopharma, China
67	Preclinical	Li-key peptide vaccine	protein subunit vaccine	Generex Biotechnology Corp., USA; Biology Institute of Shandong Academy of Sciences, China
68	Preclinical	GV-MVA-VLP vaccine platform	nonreplicating viral vector	Geovax Labs Inc., USA; Bravovax, China; Sino Biological Inc., China
69	Preclinical	Vaccine	nonreplicating viral vector; MVA-S encoded	German Center for Infection Research, Germany
70	Preclinical	Vaccine	nonreplicating viral vector; Ad5 S (GREVAX platform)	Greffex Inc., USA
71	Preclinical	gp-96 vaccine	protein subunit vaccine; gp-96 backbone	Heat Biologics Inc., USA; Zolovax Inc., USA; University of Miami Miller School of Medicine, USA
72	Preclinical	Vaxcelerate vaccine	based on self-assembling vaccine (SAV) platform	Hoth Therapeutics Inc., USA; Voltron Therapeutics Inc., USA
73	Preclinical	COVID-19 vaccine	details not known	Hualan Biological Engineering, China
74	Preclinical	IBIO-201	protein subunit vaccine; SARS-CoV-2 spike protein-based	Ibio Inc., USA
75	Preclinical	SARS-CoV-2 Virus-Like Particle	subunit protein, plant produced	Ibio Inc., USA; Beijing CC-Pharming Ltd., China
76	Preclinical	SARS-CoV-2 vaccine (injectable)	vaccine developed using Sendai virus vector	ID Pharma Co. Ltd., Japan; Fudan University, China
77	Preclinical	COVID-19 vaccine	virus suppressing factor-based vaccine	Immunemed, South Korea; Seoul National University Hospital, South Korea
78	Preclinical	Nucleic acid vaccine	plasmid DNA, needle-free delivery	Immunomic Therapeutics Inc., USA; Epivax Inc., USA; Pharmajet Inc., USA
79	Preclinical	Vaccine	protein subunit vaccine; spike-based (epitope screening)	Immunoprecise Antibodies Ltd., Canada; EVQLV Inc., USA; Litevax BV, Netherlands
80	Preclinical	Vaccine	VLP; ADDomer multiepitope display	Imophoron Ltd., UK; Bristol University’s Max Planck Centre, UK
81	Preclinical	Vaccine	saRNA vaccine	Imperial College London, UK; Maravai Lifesciences Inc., USA; Trilink Biotechnologies Inc., USA
82	Preclinical	Vaccine	developed based on recombinant vesicular stomatitis virus (rVSV) technology	International AIDS Vaccine Initiative, USA; Batavia
83	Preclinical	COVID-19 vaccine	protein subunit vaccine; outer membrane vesicle (OMV)-subunit	Intravacc, Netherlands; Epivax Inc., USA
84	Preclinical	Vaccine	DNA vaccine	Johnson & Johnson, Belgium; Beth Israel Deaconess Medical Center, USA
85	Preclinical	Vaccine	Ad26.COV2-S recombinant vaccine	Johnson & Johnson, Belgium; Biomedical Advanced Research and Development Authority (BARDA), USA; Emergent Biosolutions Inc., USA; Catalent Inc., USA
86	Preclinical	Vaccine	polypeptide vaccine	Liaoning Chengda Biotechnology, China
87	Preclinical	Vaccine	peptide-based vaccine	Ligandal Inc., USA
88	Preclinical	Vaccine	linear DNA vaccine	Linearx Inc., USA; Takis Biotech, Italy
89	Preclinical	SARS-CoV-2 vaccine	protein subunit vaccine; S-2P protein + CpG 1018	Medigen Biotechnology Corp., Taiwan; National Institutes of Health, USA
90	Preclinical	MV-014-210	live attenuated vaccine (LAV); spike protein-based	Meissa Vaccines Inc., USA
91	Preclinical	COVID-19 vaccine	replicating viral vector; replication competent VSV chimeric virus technology (VSVδG) delivering the SARS-CoV-2 Spike (S) glycoprotein	Merck & Co. Inc., USA; IAVI, USA
92	Preclinical	COVID-19 vaccine	VLP-based	Metaclipse Therapeutics, USA
93	Preclinical	Vaccine	protein subunit vaccine; oral *E. coli*-based protein expression system of S and N proteins	MIGAL Galilee Research Institute Ltd., Israel
94	Preclinical	Vaccine	details not known	Mologic Ltd., UK
95	Preclinical	COVID-19	virosome-based vaccine	Mymetics Corp., Switzerland; Mymetics BV, Switzerland; Baylor College of Medicine, USA; Texas Children’s Center for Vaccine Development, USA
96	Preclinical	COVID-19 vaccine	virosome-based vaccine	Texas Children’s Center for Vaccine Development, USA
97	Preclinical	Vaccine	peptide-based vaccine	Myneo NV, Belgium
98	Preclinical	Vaccine	nonreplicating viral vector; [E1-, E2b-, E3-] hAd5-COVID-19-spike/nucleocapsid	Nantkwest Inc., USA; Immunitybio Inc., USA
99	Preclinical	COVID-19 vaccine	based on the rBCG, genetically engineered to express selected SARS-CoV-2 proteins	Nascent Biotech Inc., USA; Manhattan Biosolutions Inc., USA
100	Preclinical	TerraCoV2	spike protein-based	Noachis Terra Inc., USA
101	Preclinical	Vaccine	protein subunit vaccine; synthetic Long peptide vaccine candidate for S and M proteins	Oncogen, Malaysia
102	Preclinical	CORVax12	co-administration of TAVO (plasmid IL-12) with a DNA-encodable version of the SARS-CoV-2 spike protein	Oncosec Medical Inc., USA
103	Preclinical	Cell-based vaccine	irradiated permissive cells (infected with a high titer virus or transfected with viral antigens)	Orgenesis Inc., USA
104	Preclinical	Vaccine	peptide-based vaccine	Ose Immunotherapeutics SA, France
105	Preclinical	VLP vaccine	protein-based vaccine	Osivax, France
106	Preclinical	COVID-19 vaccine	whole inactivated virus-based vaccine	Panacea Biotec Ltd., India
107	Preclinical	Versamune-CoV-2FC	recombinant fusion S protein-based	PDS Biotechnology Corp., USA
108	Preclinical	SARS coronavirus vaccine	receptor-binding domain of the SARS coronavirus S-protein-based	Phylex Biosciences Inc., USA
109	Preclinical	Vaccine	NSP10-based vaccine	Predictive Oncology Inc., USA
110	Preclinical	Vaccine	adenovirus vectored; spike protein-based	Reithera Srl, Italy
111	Preclinical	VLP vaccine	protein-based vaccine	Saiba AG, Switzerland
112	Preclinical	mRNA vaccine	RNA vaccine; LNP-mRNA	Sanofi Pasteur, France; Translate Bio Inc., USA
113	Preclinical	Vaccine	protein subunit vaccine	Sanofi Pasteur, France; U.S. Biomedical Advanced Research and Development Authority, USA
114	Preclinical	Vaccine	DNA vaccine	Scancell Holdings plc, UK
115	Preclinical	Vaccine	details not known	SK Bioscience Co. Ltd., South Korea
116	Preclinical	STI-6991; T-VIVA-19	recombinant fusion protein of the SARS-CoV-2 spike protein S1 domain and human IgG Fc	Sorrento Therapeutics Inc., USA; Smartpharm Therapeutics Inc., USA
117	Preclinical	OraPro-COVID-19	nonreplicating viral vector; oral Ad5 S	Stabilitech Biopharma Ltd., UK
118	Preclinical	Vivagel (SPL-7013)	astodrimer sodium-based	Starpharma Ltd., Australia
119	Preclinical	Vaccine	VSV-receptor binding domain vaccine	Sumagen, South Korea; International Vaccine Institute, South Korea
120	Preclinical	VLP vaccine	recombinant protein vaccine	Sysvax Inc., China
121	Preclinical	COVID-eVax	DNA-based; encodes a part of viral spike protein	Takis Srl, Italy; Rottapharm Biotech Srl, Italy
122	Preclinical	Vaccine	bivalent COVID-19 vaccine	Tevogen Bio Inc., USA
123	Preclinical	mRNA vaccine	RNA vaccine	Tongji University, China; Stemirna Therapeutics Co. Ltd., China
124	Preclinical	COVID-19 vaccine	live replicating virus vaccine	Tonix Pharmaceuticals Holding Corp., USA; Kansas State University, USA
125	Preclinical	TNX-1800	replicating viral vector; horsepox vector expressing S protein	Tonix Pharmaceuticals Holding Corp., USA; University of Alberta, USA; Fujifilm Diosynth Biotechnologies, USA; Southern Research, USA
126	Preclinical	PolyPEPI-SCoV-2	consists of 10 different, 30-amino acid long synthetic peptides	Treos Bio Ltd., UK
127	Preclinical	Vaccine	details not known	Tulane University, USA
128	Preclinical	Vaccine	VLP vaccine	Ufovax Inc., USA
129	Preclinical	Vaccine	replicating viral vector; influenza vector expressing RBD	University of Hong Kong, Hong Kong
130	Preclinical	Measles vector-based vaccine (PittCoVacc)	replicating viral vector; measles vector	University of Pittsburgh, USA; Themis Biosciences Inc., Austria; Coalition for Epidemic Preparedness Innovations, Norway; Pasteur Institute, France; Merck & Co. Inc., USA
131	Preclinical	Protein subunit vaccine	molecular clamp stabilized spike protein	University of Queensland, Australia; Glaxosmithkline plc., UK; Seqirus GmbH, UK; Dynavax Technologies Corp., USA
132	Preclinical	SARS-CoV-2 vaccine	VLPs peptides/whole virus	University of Sao Paulo, Brazil
133	Preclinical	Vaccine	protein subunit vaccine; adjuvanted microsphere peptide	University of Saskatchewan, Canada
134	Preclinical	Ixiaro	inactivated + CpG 1018	Valneva SE, France; Dynavax Technologies Corp., USA
135	Preclinical	Pepticrad vaccine	nonreplicating viral vector; adenovirus-based + HLA-matched peptides	Valo Therapeutics Ltd., Finland
136	Preclinical	Vaccine	nanoparticle-based delivery system	Vault Pharma Inc., USA; University of California, Los Angeles, USA; Northern Arizona University, USA
137	Preclinical	COVID-19 oral vaccine	nonreplicating viral vector; oral recombinant vaccine for mucosal and systemic immune responses	Vaxart Inc., USA; Emergent Biosolutions Inc., USA
138	Preclinical	Peptide vaccine	protein subunit vaccine	Vaxil Bio Ltd., Canada
139	Preclinical	Vaccine	enveloped virus-like particle vaccine	VBI Vaccines Inc., USA; National Research Council of Canada, Canada
140	Preclinical	Vaxipatch vaccine	dermal patch with a metal microneedle array for delivery	Verndari Inc., USA
141	Preclinical	Vaccine	spike protein-based	Vir Biotechnology Inc., USA; Glaxosmithkline plc., UK
142	Preclinical	Vaccine	spike protein-based	Viravaxx AG, Austria; Medical University of Vienna, Austria
143	Preclinical	Vaccine	spike protein-based	Walter Reed Army Institute of Research, USA; U.S. Army Medical Research and Development Command, USA
144	Preclinical	COVID-19 XWG-03	protein subunit vaccine; COVID-19 XWG-03 truncated S (spike) proteins	Xiamen Innovax Biotech Co. Ltd., China; Glaxosmithline plc., UK; Xiamen University, China
145	Preclinical	Vaccine	protein subunit vaccine; recombinant protein	Yisheng Biopharma Co. Ltd., China
146	Preclinical	ZIP-1642	mRNA vaccine	Ziphius Therapeutics NV, Belgium; Ghent University, Belgium

For further information visit the following links: https://clinicaltrials.gov & https://www.bioworld.com/COVID19products#vac1.

During the vaccine candidate development against SARS-CoV-2, one may have to consider the possibility of antibody-dependent enhancement (ADE) triggering in vaccinated individuals where instead of mounting protection against the virus infection the virus-bound antibody bind to the host cell receptors to facilitate the cellular entry of the virus. Activation of ADE has been observed in vaccines against several diseases, e.g., Ebola, HIV, Dengue, feline coronavirus, etc (Takada and Kawaoka, 2003; Halstead, 2017; Takano et al., 2019). Human and rodent antibodies produced against the SARS-CoV S protein also shown to induce ADE *in vitro* (Liu et al., 2019). However, ADE was not observed in several pre-clinical studies done in rhesus monkeys using a SARS-CoV vaccine (Luo et al., 2018). Besides, in a pre-clinical study using an inactivated SARS-CoV-2 vaccine did not show any evidence of ADE (Gao Q. et al., 2020).

### Therapeutics

SARS‐CoV‐2 does not use receptors that are utilized by other coronaviruses, for example, APN (aminopeptidase N; used by HCoV-229E), DPP4 (dipeptidyl peptidase 4; used by MERS-CoV), or O-acetylated sialic acid receptor (used by HCoV-OC43 and HCoV-HKU1) (Yeager et al., 1992; Krempl et al., 1995; Raj et al., 2013; Huang et al., 2015). It uses the human ACE2 cell receptor to enter the host cell, similar to SARS-CoV and HCoV-NL63 (Hofmann et al., 2005; Ge et al., 2013; Wrapp et al., 2020). So, soluble human ACE2 protein can also be a potential competitor for the ACE2 cell surface receptor, but it can only be achieved when the gene expression of soluble ACE2 is higher than the gene expression of cell surface ACE2 receptor. However, an increase in the concentration of soluble ACE2 in blood found to be associated with chronic cardiac dysfunction (Epelman et al., 2008; Epelman et al., 2009; Ortiz-Pérez et al., 2013). SARS-CoV was found to downregulate ACE2 by binding to it by its spike protein and inflicting severe lung damage (Kuba et al., 2005). Therefore, overexpressed soluble ACE2 may help in neutralizing SARS-CoV-2 by competitively binding to it and free the cellular ACE2 to perform its normal function. A recombinant human ACE2 (APN01) was found to decrease the levels of angiotensin II and plasma IL-6 in different patients diagnosed with ARDS (acute respiratory distress syndrome) may also be utilized for inhibiting SARS-CoV-2 from accessing cellular ACE2 receptor (Zhang et al., 2020a). Soluble human ACE2 protein was shown to bind SARS-CoV with an affinity close to the affinities of monoclonal antibodies and blocks the virus from accessing cellular ACE2 receptor in cell culture (Li et al., 2003; Sui et al., 2004). Interestingly, membrane-anchored metalloproteinase ADAM17 cleaves ACE2 to release the soluble ACE2 domain, which was predicted to have some adverse effects on the heart (Jiang et al., 2014).

Another strategy is to develop anti-ACE2 antibodies that would bind to the human ACE2 protein and block this viral entry, as was shown in SARS-CoV (Li et al., 2003). Unfortunately, there are problems with generating antibodies or protein fragments against the cellular ACE2 as it plays several important roles in controlling cardiovascular diseases including heart attack, diabetes, kidney problems, high blood pressure, etc. Therefore, inactivating the cellular ACE2 receptor is probably not a viable solution.

Alternatively, an ACE2-Fc fusion protein can also increase the lifespan of the soluble ACE2 protein in circulation and inhibit the virus from accessing the cellular ACE2 receptor. Similarly, in a study, the extracellular ACE2 domain fused to the human IgG1 domain was shown to neutralize the SARS-CoV *in vitro* (Gu et al., 2016), which shows that the use of ACE2-Fc could be a viable solution to block SARS-CoV-2 from infecting human cells. However, this strategy may induce ADE and therefore a thorough investigation is needed to eliminate any adverse effects. The spike protein RBD could also be attached to a human IgG Fc fragment to increase its immunogenicity and stability (Zhang et al., 2009; Li et al., 2011; Du et al., 2013b), as was done in MERS-CoV (Du et al., 2013a). The MERS-CoV spike protein RBD-Fc fusion was found useful in blocking viral cell surface receptor from accessing it by the virus and also stimulated the host immune response against the viral protein domain in mice (Du et al., 2013a). Here one has to consider the mutation of the Fc domain that eliminates its cellular Fc receptor (FcγR) binding ability and triggering of cytotoxic effects (Wang et al., 2018; Kang and Jung, 2019). The binding of the Fc region to FcγR would activate immune cells to trigger the ADCC pathway and release proinflammatory cytokines, which may lead to cytokine storm (Wang et al., 2018). Therefore, the Fc fusion strategy requires a thorough investigation of toxicity and efficacy, followed by the engineering of the Fc fragment for immune silencing and increasing effectiveness (Kang and Jung, 2019).

The other alternative strategy would be to generate antibodies or protein-fragments that would bind to the virus itself and protect the cellular ACE2 receptor from binding the virus (Jiang et al., 2020). If a protein or peptide fragment that can mimic the binding domain of ACE2 cell receptor and induce similar changes in conformation, as the receptor likely does, then also it can compete with the ACE2 cell receptor. Recently a 23-mer peptide designed from the ACE2 α1 helix has shown a specific binding affinity toward RBD of S protein from SARS-CoV-2, which shows that the development of a peptide-based therapeutics is possible that blocks of this virus interaction with human ACE2 and protecting the cell from virus entry (Zhang G. et al., 2020).

A recent report has shown that murine polyclonal antibodies generated against SARS-CoV spike protein were capable enough to inhibit spike protein-mediated cellular entry of SARS-CoV-2 (Walls et al., 2020). Also, a human monoclonal antibody (47D11), which interacts with a conserved epitope on RBD of spike protein, was found to cross-neutralize with both SARS-CoV-2 and SARS-CoV (Wang et al., 2020b). Another antibody having neutralizing property (antibody CR3022) previously isolated from the SARS-CoV infected patient was found to interact with the S protein RBD of SARS-CoV-2 at a site different from the ACE2 binding site indicating cross-reactivity of the antibody for having similar structural regions on the spike proteins of both the viruses (Yuan et al., 2020).

SARS-CoV-2 nucleocapsid protein (N) is another vital protein having several critical roles, including viral genome replication, transcription, etc., and therefore is an attractive drug target. Recently a 3D structure (x-ray crystallography) of the amino-terminal RNA-binding domain of this virus N protein has been elucidated, indicating drug targets (Kang et al., 2020). Broad-spectrum antiparasitic drug nitazoxanide has been shown to inhibit the expression of nucleocapsid protein in MERS-CoV and other coronaviruses (Rossignol, 2016). Nitazoxanide also found to suppress proinflammatory cytokines, including IL-6 in mice (Rossignol, 2016). The viral M protein is also highly conserved in evolution among different species (Neuman et al., 2011), and hence, may also be used as a candidate for developing the SARS-CoV-2 therapeutics (**Table 2**).

**Table 2:** Ongoing repurposed drug/therapeutic molecule development by different organizations against COVID-19 that are at different phases of clinical trials (updated on July 25, 2020).

No.	Clinical stage	Drug name	Other disease targets	Mode of action	Organization/Company
1	Compassionate use (phase II/III)	Ifenprodil (NP-120)	peripheral circulatory disorders; idiopathic pulmonary fibrosis	inhibitor of the N-methyl-D-aspartate receptor	Algernon Pharmaceuticals Inc., Canada; Nash Pharmaceuticals, Canada
2	Compassionate use (phase II/III)	DAS-181	influenza; parainfluenza	removes sialic acid from the respiratory cells	Ansun Biopharma Inc., USA
3	Compassionate use (phase II)	Piclidenoson	rheumatoid arthritis	antagonism of adenoside A3 receptors; induce anti-inflammatory effects	Can-Fite Biopharma Ltd., Israel; Lewis Katz School of Medicine at Temple University, USA
4	Compassionate use (phase III)	Siltuximab (Sylvant)	multicentric Castleman’s disease	monoclonal antibody that binds to IL-6	Eusa Pharma Inc., UK
5	Compassionate use (phase III)	Tocilizumab (Actemra)	rheumatoid arthritis; systemic juvenile idiopathic arthritis	monoclonal antibody against the IL-6 receptor	Genentech Inc., USA
6	Compassionate use (phase III)	Lenzilumab	chronic myelomonocytic leukemia; juvenile myelomonocytic leukemia	humanized monoclonal antibody that targets CSF2/GM-CSF	Humanigen Inc., USA
7	Compassionate use (phase II)	IC14	acute lung injury; motor neuron disease	monoclonal antibody; CD14 antigen inhibitor	Implicit Bioscience Ltd., USA
8	Compassionate use	Namilumab (IZN-101)	ankylosing spondylitis	monoclonal antibody; GM-CSF antagonist	Izana Bioscience Ltd., UK
9	Compassionate use (phase II/III)	Mavrilimumab	rheumatoid arthritis	monoclonal antibody that inhibits human GM-CSF-receptor	Kiniksa Pharmaceuticals Ltd., Bermuda
10	Compassionate use (phase II/III)	Giapreza	hypotension	Angiotensin type 1 receptor agonist	La Jolla Pharmaceutical Co., USA
11	Compassionate use (phase I/II)	Organicell Flow	regenerative therapy	acellular product derived from human amniotic fluid; suppressor of cytokine activation	Organicell Regenerative Medicine Inc., USA
12	Compassionate use	Conestat alfa (Ruconest)	hereditary angioedema	complement component C1r, C1s inhibitor	Pharming Group, Netherlands
13	Compassionate use (phase II)	PLX cell product candidates	cancer	placenta-based cell therapy	Pluristem Therapeutics Inc., Israel; Charite’ University of Medicine Berlin, Germany
14	Compassionate use	Allorx stem cells	anti-aging	adult mesenchymal stem cell (MSC)-based therapy	Vitro Diagnostics Inc., USA; Global Institute of Stem Cell Therapy and Research Inc. (Giostar), USA
15	Emergency use authorization	Bemsivir (generic remdesivir)	ebola	viral RNA polymerase inhibitor	Beximco Pharmaceuticals Ltd., Bangladesh; Hetero Labs Ltd., India; Mylan NV, USA
16	Emergency use authorization (phase III, expanded access, benefit; approved in EU)	Remdesivir (Veklury)	ebola	viral RNA polymerase inhibitor	Gilead Sciences Inc., USA; Cipla Ltd., India; Hetero Labs Ltd., India; Dr. Reddy’s Laboratories Inc., India
17	Emergency use authorization (submitted)	MSCs	regenerative therapy for various injuries	mesenchymal stromal cell-based therapy	Predictive Biotech, USA
18	Emergency use authorization - REVOKED (phase III, no benefit)	Chloroquine/hydroxychloroquine (Plaquenil)	malaria	increases lysosomal pH; membrane fusion inhibitor	Sanofi SA, France; Amneal Pharmaceuticals Inc., USA; Rising Pharma Holdings Inc., USA; University of Minnesota, USA; Sandoz Inc., Germany; Bayer AG, Germany; University of Washington, USA; Patient-Centered Outcomes Research Institute (PCORI), USA; Certara Inc., USA; Progenabiome LLC, USA
19	Expanded access (phase II)	Eculizumab (Soliris)	paroxysmal nocturnal hemoglobinuria; atypical hemolytic uremic syndrome; neuromyelitis optica	complement C5 inhibitor	Alexion Pharmaceuticals Inc., USA
20	Expanded access (phase III)	Inopulse	pulmonary arterial hypertension	vasodilator nitric oxide decreases pressure in the pulmonary arteries; improves oxygination	Bellerophon Therapeutics Inc., USA
21	Expanded access	CAP-1002	Duchenne muscular dystrophy; myocardial infarction	cardiosphere-derived cell replacement therapy	Capricor Therapeutics Inc., USA
22	Expanded access (phase II/III)	Ruxolitinib (Jakafi)	myelofibrosis	Janus kinase-1/2 inhibitor	Incyte Corp., USA; Novartis AG, Switzerland
23	Expanded access (phase II/III)	Remestemcel-L	acute graft versus host disease (aGVHD)	culture-expanded mesenchymal stem cell replacement therapy	Mesoblast Ltd., Australia
24	Expanded access (phase II/III)	Opaganib (Yeliva)	cancer	inhibitor of the enzyme sphingosine kinase 2	Redhill Biopharma Ltd., Israel; Apogee Biotechnology Corp., USA
25	Expanded access	Genosyl DS	pulmonary arterial hypertension	nitric oxide delivery system; improves oxygination	Vero Biotech LLC, USA
26	Phase IV	Danoprevir (Ganovo) + ritonavir	hepatitis C; AIDS	viral protease inhibitor	Ascletis Pharma Inc., China
27	Phase IV	Berberine	diabetes; hyperlipidemia; high blood pressure; gastrointestinal infections	AMP-activated protein kinase (AMPK) activator; α-glucosidase inhibitor	Chinese Medical Association, China
28	Phase IV	Irbesartan (DMX-200)	hereditary angioedema	complement component C1r, C1s inhibitor	Dimerix Ltd., Australia
29	Phase IV	Eritoran	sepsis	endotoxin inhibitor; lipid A inhibitor; toll-like receptor 4 antagonist	Eisai Co. Ltd., Japan
30	Phase IV	Interferon-beta-1a (Traumakine)	multiple sclerosis	immunostimulants; interferon beta-1a replacements	Faron Pharmaceuticals, Finland
31	Phase IV	Bivalirudin (Angiomax)	acute coronary syndromes; hrombosis	thrombin inhibitor	Hamad Medical Corp., Qatar
32	Phase IV	Cyclosporine	rheumatoid arthritis; psoriasis; Crohn’s disease; organ rejection	calcineurin inhibitor; immunosuppressant	Instituto de Investigacion Sanitaria de la Fundacion Jimenez Diaz, Spain; University of Pennsylvania, USA
33	Phase IV	N-acetylcysteine	bronchiectasis; chronic obstructive pulmonary disease; cystic fibrosis	antioxidant	Memorial Sloan Kettering Cancer Center, USA; Cambridge Health Alliance, USA; Mashhad University of Medical Sciences, Iran; Shuguang Hospital, China; Hubei Hospital of Traditional Chinese Medicine, China; Jingmen No. 1 People’s Hospital, China; Tongji Hospital, China
34	Phase IV	Interferon beta-1a (Rebif)	multiple sclerosis	immunostimulant; interferon beta-1a replacement	Merck Group, Germany; French Institut National de la Sante et de la Recherche Medicale (INSERM), France
35	Phase IV	Ebastine	allergic conjunctivitis; allergic rhinitis; urticaria	Histamine H1 receptor antagonist	Mianyang Central Hospital, China; Wuhan Red Cross Hospital, China; West china Hospital of Sichuan University, China
36	Phase IV	Sargramostim (Leukine)	acute radiation syndrome; bone marrow disorders; neutropenia	granulocyte stimulant; haematopoiesis stimulants; neutrophil stimulant	Partner Therapeutics Inc., USA
37	Phase IV	Umifenovir (Arbidol)	influenza	membrane fusion inhibitor	Pharmstandard, Russia
38	Phase IV	Valsartan	heart failure; hypertension; postmyocardial infarction	angiotensin type 1 receptor antagonists	Radboud University, Netherlands
39	Phase IV	Baloxavir marboxil (Xofluza)	influenza	endonuclease inhibitors	Roche Holding AG, Switzerland; The First Affiliated Hospital of Zhejiang University Medical School, China
40	Phase IV	Carrimycin	cancer	50S ribosomal subunit inhibitor	Shenyang Tonglian Group Co. Ltd., China
41	Phase III (no benefit)	Lopinavir/ritonavir (Kaletra/Aluvia)	AIDS	viral protease inhibitor	Abbvie Inc., USA
42	Phase III	Dornase alfa (Pulmozyme)	cystic fibrosis	deoxyribonuclease 1 stimulant	Acibadem University, Turkey; The Scientific and Technological Research Council of Turkey; University College, London, UK; Feinstein Institute for Medical Research, USA; Cold Spring Harbor Laboratory, USA; Northwell Health, USA; Fondation Ophtalmologique Adolphe de Rothschild, France; University Hospital, Strasbourg, France; Hospital Center Régional Metz-Thionville, France; University of Missouri-Columbia, USA; Boston Children’s Hospital, USA; Brigham and Women’s Hospital, USA; University of South Alabama, USA
43	Phase III	Ravulizumab (Ultomiris)	paroxysmal nocturnal haemoglobinuria	complement C5 inhibitor	Alexion Pharmaceuticals Inc., USA
44	Phase III	Tigerase (dornase alfa biosimilar)	cystic fibrosis	deoxyribonuclease 1 stimulant	AO Generium, Russia
45	Phase III	ASC-09 + ritonavir (oral tablet)	HIV	cytochrome P 450 enzyme system inhibitor; HIV protease inhibitor	Ascletis Pharma Inc., China
46	Phase III	Almitrine	chronic obstructive pulmonary disease	agonist of peripheral chemoreceptors located on the carotid bodies	Assistance Publique - Hôpitaux de Paris, France; Centre Hospitalier de Chartres, France
47	Phase III	Dapagliflozin (Farxiga)	sodium-glucose transporter 2 inhibitor	cardiovascular disorders; diabetes mellitus	Astrazeneca, UK
48	Phase III	Chloroquine + interferon beta-1b	malaria; multiple sclerosis	membrane fusion inhibitor; immunostimulant	Bayer Inc., Germany; Population Health Research Institute, Canada
49	Phase III	Levilimab	rheumatoid arthritis	human antibody inhibitor of IL-6 receptor	Biocad, Russia
50	Phase III	NK1R+ MSC	myocardial infarction; left ventricular dysfunction	cell replacement	Biocardia Inc., USA; University of Health Sciences Lahore, Pakistan
51	Phase III	Rivaroxaban	deep vein thrombosis; pulmonary embolism	factor Xa inhibitor	Charite University, Germany; Deutsches Zentrum für Herz-Kreislauf-Forschung, Germany; Bayer AG, Germany
52	Phase III	Methylprednisolone	multiple sclerosis	immunosuppressants; steroid receptor agonists	Chinese research sponsors, China; University of Oxford, UK; University of Chile, Chile
53	Phase III	Ciclesonide (Alvesco)	allergic rhinitis; asthma	glucocorticoid receptor agonists; immunosuppressants	Covis Pharma, Switzerland
54	Phase III	Pacritinib	myelofibrosis	Fms-like tyrosine kinase 3 inhibitor; Janus kinase-2 inhibitor	CTI Biopharma Corp., USA
55	Phase III	Baricitinib (Olumiant)	rheumatoid arthritis	AAK1 inhibitor; JAK-STAT pathway inhibitor; endocytosis inhibitor	Eli Lilly and Co., USA; Incyte Corp., USA
56	Phase III	Radiation therapy	cancer	breaks DNA of cancer cells	Emory University, USA; others
57	Phase III	ENU-200	viral infection	glycoprotein inhibitors; peptide hydrolase inhibitors	Ennaid Therapeutics LLC, USA
58	Phase III (approved in India)	Favipiravir (Avigan)	influenza	viral RNA polymerase inhibitor	Fujifilm Holdings Corp., Japan; Fujifilm Toyama Chemical Co. Ltd., Japan; Medivector Inc., USA; Zhejiang Hisun Pharmaceutical Co. Ltd., China; Sihuan Pharmaceutical Holdings Group Ltd., China; Genentech Inc., USA; Appili Therapeutics Inc., Canada; Glenmark Pharmaceuticals Ltd., India; Dr. Reddy’s Laboratories, India
59	Phase III	Losmapimod	facioscapulohumeral muscular dystrophy	DUX4 protein inhibitor; P38 mitogen-activated protein kinase inhibitor	Fulcrum Therapeutics Inc., USA
60	Phase III	Alteplase (tissue plasminogen activator)	catheter thrombosis; myocardial infarction; pulmonary embolism	fibrinolytic agents; plasminogen activator stimulants	Genentech Inc., USA; University of Colorado Denver, USA; Negovsky Reanimatology Research Institute, Russia; Sklifosovsky Institute of Emergency Care, Russia
61	Phase III	Emtricitabine/tenofovir (Truvada)	AIDS	reverse transcriptase inhibitor	Gilead Sciences Inc., USA
62	Phase III	Tacrolimus	eczema; psoriasis; allogeneic organ transplant	bone morphogenetic protein receptor type II modulator; cytokine inhibitor; T cell activation inhibitor	Hospital Universitari de Bellvitge, Spain; Institut d’Investigació Biomèdica de Bellvitge, Spain
63	Phase III	IMM-101	cancer	dendritic cell stimulant; immunostimulant	Immodulon Therapeutics Ltd., UK; Biocan Rx, Canada; Canadian Cancer Trials Group; Canadian Cancer Society Research Institute; Atgen Canada Inc.; Canadian Centre for Applied Research in Cancer Control; Ontario Institute for Cancer Research, Canada
64	Phase III	Bacmune (MV-130)	respiratory tract infections	immunostimulant	Immunotek, USA; Bioclever 2005 SL, Spain
65	Phase III	Darunavir/cobicistat (Prezcobix)	AIDS	cytochrome P 450 enzyme system inhibitor; HIV protease inhibitor	Johnson & Johnson, USA
66	Phase III	Hydroxychloroquine and other lupus therapies	malaria; lupus	increases lysosomal pH; membrane fusion inhibitor; immunosuppressant	Lupus Therapeutics, USA
67	Phase III	Colchicine	familial mediterranean fever; gout	tubulin polymerisation inhibitor	Montreal Heart Institute, Canada
68	Phase III	Doxycycline	exanthema; acne	30S ribosomal subunit inhibitor	Nantes University Hospital, France
69	Phase III	Famotidine	gastritis; peptic ulcer	histamine H2 receptor antagonist	Northwell Health, USA; Cold Spring Harbor Laboratory, USA
70	Phase III	Hydroxychloroquine	malaria	autophagy inhibitor; phospholipase A2 inhibitor	Novartis, Switzerland
71	Phase III	Canakinumab (Ilaris)	systemic juvenile idiopathic arthritis; active Still’s disease	Interleukin 1 beta inhibitor	Novartis, Switzerland
72	Phase III	Octagam 10%	idiopathic thrombocytopenic purpura; Immunodeficiency disorder	immunostimulant	Octapharma USA Inc., USA
73	Phase III	CD24Fc	graft-versus host disease (GVHD)	interleukin 1 beta inhibitor; interleukin 6 inhibitor; tumour necrosis factor alpha inhibitor	Oncoimmune Inc., USA
74	Phase III	Azithromycin (Zithromax)	bacterial infections; acute sinusitis	50S ribosomal subunit inhibitor	Pfizer Inc., USA
75	Phase III	REGN-COV2 (REGN-10933 + REGN-10987)	viral infection	antibody; virus internalisation inhibitor	Regeneron Pharmaceuticals Inc., USA
76	Phase III	Dactolisib (RTB-101)	cancer	phosphatidylinositol 3 kinase (PI3K) inhibitor; mammalian target of rapamycin (mTOR) inhibitor	Restorbio Inc., USA; Adicet Bio Inc., USA
77	Phase III	Bucillamine	gout; rheumatoid arthritis	immunomodulator; xanthine oxidase inhibitor	Revive Therapeutics Ltd., Canada; Novotech Pty Ltd., Australia
78	Phase III	Oseltamivir (Tamiflu)	influenza	neuraminidase inhibitor; exocytosis inhibitor	Roche Holding AG, Switzerland
79	Phase III	Tocilizumab (Actemra)	rheumatoid arthritis	IL-6 receptor inhibitor	Roche Holding AG, Switzerland
80	Phase III	Nitazoxanide (NT-300)	antiparasitic	nucleocapsid protein inhibitor; suppress IL-6 production	Romark Laboratories LC, USA
81	Phase III	Enoxaparin (Lovenox)	deep vein thrombosis; embolism; myocardial infarction	factor Xa inhibitor; thrombin inhibitor	Sanofi, France
82	Phase III	Dipyridamole	stroke; transient ischaemic attack	platelet aggregation inhibitor	UConn Health, USA; University of Michigan, USA; Rutgers University, USA; Boehringer Ingelheim GmbH, Germany
83	Phase III	Tradipitant	atopic dermatitis	neurokinin-1 receptor (NK-1R) antagonist	Vanda Pharmaceuticals Inc., USA; University of Illinois at Chicago, USA
84	Phase II/III	ABX-464	AIDS; rheumatoid arthritis; ulcerative colitis	immunostimulant; rev gene product inhibitor; RNA cap-binding protein modulator	Abivax, France
85	Phase II/III	Multistem	neurological, inflammatory, cardiovascular diseases	multipotent adult progenitor cell therapy	Athersys Inc., USA
86	Phase II/III	BDB-001	tumor	immunomodulator; toll-like receptor 7 agonist; toll-like receptor 8 agonist	Beijing Defengrei Biotechnology Co., China
87	Phase II/III	BC-007	dilated cardiomyopathy; chronic fatigue syndrome	immunomodulators; virus replication inhibitor	Berlin Cures Holding AG, Germany
88	Phase II/III	Vazegepant	migraine	calcitonin gene-related peptide receptor antagonist	Biohaven Pharmaceutical Holding Co. Ltd., USA
89	Phase II/III	Sarconeos (BIO-101)	duchenne muscular dystrophy	proto-oncogene protein c-mas-1 agonist	Biophytis SA, France
90	Phase II/III	Lactoferrin	Crohn’s disease	chelating agent; immunomodulator	Cairo University, Egypt; National Research Center, Egypt; Egyptian Military Medical Services
91	Phase II/III	Sofosbuvir, daclatasvir, hydroxychloroquine; sofosbuvir, ribavirin	hepatitis C; malaria	virus replication inhibitor; membrane fusion inhibitor	Cairo University, Egypt; Tanta University, Egypt
92	Phase II/III	Ambrisentan	pulmonary arterial hypertension	endothelin A receptor antagonist	Cambridge University Hospitals, UK; NHS Foundation Trust, UK
93	Phase II/III	Dociparstat sodium	acute myeloid leukaemia; pancreatic cancer	cathepsin G inhibitor; chemokine CXCL12 inhibitor	Chimerix Inc., USA
94	Phase II/III	PRO-140 (leronlimab)	AIDS	binds to CCR5 receptor to block HIV; membrane fusion inhibitor	Cytodyn Inc., USA
95	Phase II/III	EB-05	rheumatoid arthritis	toll-like receptor 4 antagonist	Edesa Biotech Inc., Canada; Novimmune SA, Switzerland
96	Phase II/III	Nafamostat mesylate	pancreatitis	serine protease TMPRSS-2 inhibitor; membrane fusion inhibitor	Ensysce Biosciences Inc., USA
97	Phase II/III	EDP-1815	atopic dermatitis; psoriasis	Immunomodulator	Evelo Biosciences Inc., USA; Cambridge University Hospitals NHS Foundation Trust, UK
98	Phase II/III	Levamisole	parasitic worm infections	Immunomodulator	Fasa University of Medical Sciences, Iran; Ain Shams University, Egypt; Cairo University, Egypt
99	Phase II/III	Pamrevlumab	idiopathic pulmonary fibrosis; pancreatic cancer	connective tissue growth factor inhibitor	Fibrogen Inc., USA
100	Phase II/III	Bevacizumab	cancer	Angiogenesis inhibitors; vascular endothelial growth factor A inhibitor	Genentech Inc., USA
101	Phase II/III	Atazanavir; daclatasvir; sofosbuvir; favipiravir	hepatitis C, AIDS, ebola	viral protein/protease/replicase inhibitor	Hospital do Coracao, Brazil
102	Phase II/III	IFX-1	sepsis; systemic inflammatory response syndrome	complement C5a inhibitor; inflammation mediator modulator	Inflarx, Germany
103	Phase II/III	Cannabidiol	fragile X syndrome; epilepsy; pain; insomnia; anxiety	antioxidant; cannabinoid receptor CB1/CB2 inverse agonists; serotonin 1 receptor modulator	Innocan Pharma Corp., Israel; Ramot at Tel Aviv University, Israel; University of Sao Paulo, Brazil
104	Phase II/III	Candesartan	hypertension	angiotensin receptor blocker	Medical University of Vienna, Austria
105	Phase II/III	Ivermectin	parasitic infections	viral protein maturation inhibitor	Medincell SA, France; Merck, USA
106	Phase II/III	Previfenon	heart and brain disease	reduce inflammation	Melisa Institute Genomics & Proteomics Research, Chile; Universidad Australia
107	Phase II/III	NA-831 + atazanavir + dexamethasone	alzheimer’s disease; AIDS; rheumatoid arthritis	HIV protease inhibitor; immunosuppressant	Neuroactiva Inc., USA
108	Phase II/III	Aviptadil (RLF-100)	pulmonary sarcoidosis	vasoactive intestinal peptide receptor agonist	Neurorx Inc., USA; Relief Therapeutics Holding SA, Switzerland
109	Phase II/III (benefit)	Dexamethasone	skin diseases; asthma; cancer; rheumatoid arthritis	glucocorticoid receptor agonist; immunosuppressant	Oxford University, UK
110	Phase II/III	PTC-299	acute myeloid leukaemia	dihydroorotate dehydrogenase inhibitor	PTC Therapeutics Inc., USA
111	Phase II/III (no benefit, halted)	Sarilumab (Kevzara)	rheumatoid arthritis	IL-6 receptor inhibitor	Regeneron Pharmaceuticals Inc., USA; Sanofi SA, France
112	Phase II/III	Olokizumab + RPH-104	rheumatoid arthritis; pain	IL-6 inhibitor; interleukin 1 beta inhibitor	R-Pharm JSC, Russia; Cromos Pharma LLC
113	Phase II/III	Emapalumab (Gamifant)	haemophagocytic lymphohistiocytosis	interferon gamma inhibitor	Swedish Orphan Biovitrum, Sweden
114	Phase II/III	Anakinra (Kineret)	rheumatoid arthritis	IL-1 receptor inhibitor	Swedish Orphan Biovitrum, Sweden
115	Phase II/III	RESP-301	influenza	antiviral; prevent membrane fusion; virus replication inhibitor	Thirty Respiratory Ltd., UK
116	Phase II/III	Losartan	diabetic nephropathies; heart failure; hypertension	angiotensin type 1 receptor antagonist	University of Minnesota, USA
117	Phase II/III	Generic hydroxychloroquine	malaria	autophagy inhibitor; phospholipase A2 inhibitor	Walter and Eliza Hall Institute of Medical Research, Australia; Iqvia Inc., USA
118	Phase II	MRx-4DP0004	asthma	immunomodulator	4D Pharma plc, UK
119	Phase II	Masitinib	mastocytosis; cancer	tyrosine kinase inhibitor	AB Science, France
120	Phase II	Ibrutinib	chronic lymphocytic leukaemia; graft-versus-host disease	tyrosine kinase inhibitor	Abbvie Inc., USA; Janssen Research & Development LLC, USA
121	Phase II	LY-3819253 (LY-CoV555)	viral infection	human antibody inhibitor of cell entry	Abcellera Biologics Inc., Canada; Eli Lilly and Co., USA
122	Phase II	ATI-450	rheumatoid arthritis	MAP-kinase-activated kinase 2 inhibitor	Aclaris Therapeutics Inc., USA; University of Kansas Medical Center, USA
123	Phase II	Epoprostenol (Ventoprost)	pulmonary hypertension	epoprostenol receptor agonist; platelet aggregation inhibitor	Aerogen Pharma Ltd., Ireland; Ohio State University, USA
124	Phase II	Razuprotafib	diabetic macular oedema; diabetic retinopathy; ocular hypertension	angiopoietin modulator; receptor-like protein tyrosine phosphatase inhibitor; TIE-2 receptor agonist	Aerpio Pharmaceuticals Inc., USA; Quantum Leap Healthcare Collaborative, USA
125	Phase II	Apilimod (LAM-002A)	non-Hodgkin’s lymphoma	phosphatidylinositol 3 kinase inhibitor	AI Therapeutics Inc., USA; Yale University, USA; Quantitative Biosciences Institute at UC San Francisco, USA
126	Phase II	Vadadustat	anaemia	hypoxia-inducible factor-proline dioxygenase inhibitor	Akebia Therapeutics Inc., USA
127	Phase II	Rapamycin (Sirolimus)	coronary artery restenosis; lymphangioleiomyomatosis; renal transplant rejection; fibroma	immunosuppressant; methylmalonyl CoA mutase stimulant; MTOR protein inhibitor; T lymphocyte inhibitor	Alexandria University, Egypt; University of Texas at San Antonio
128	Phase II	ANG-3777	acute kidney injury; pneumonia; renal failure	hepatocyte growth factor stimulant	Angion Biomedica Corp., USA
129	Phase II	APN-01	cancer; diabetic nephropathies; heart failure; hypertension	ACE stimulant; virus internalisation inhibitor	Apeiron Biologics, Austria
130	Phase II	AT-001	rheumatoid arthritis	immunomodulator	Applied Therapeutics Inc., USA
131	Phase II	Cilastatin (MetaBlok)	cancer; sepsis; acute kidney injury	dipeptidase inhibitor	Arch Biopartners Inc., Canada
132	Phase II	Ramelteon	insomnia	melatonin MT1/MT2 receptor agonist	Associacao Fundo de Incentivo a Pesquisa, Brazil
133	Phase II	Acalabrutinib (Calquence)	chronic lymphocytic leukaemia	tyrosine kinase inhibitor	Astrazeneca, UK
134	Phase II	MEDI-3506	atopic dermatitis; diabetic nephropathies	IL-33 inhibitor	Astrazeneca, UK
135	Phase II	AT-527	hepatitis C	hepatitis C virus NS 5 protein inhibitor	Atea Pharmaceuticals Inc., USA
136	Phase II	ATYR-1923	pulmonary sarcoidosis	neuropilin-2 modulator	Atyr Pharma Inc., USA
137	Phase II	Co-trimoxazole	bacterial infection	tetrahydrofolate dehydrogenase inhibitor	Bangabandhu Sheikh Mujib Medical University, Bangladesh; Anwar Khan Modern Medical College and Hospital, Bangladesh; Mugda Medical College and Hospital, Bangladesh
138	Phase II	Ribavirin (Virazole)	hepatitis C	nucleic acid inhibitor	Bausch Health Cos. Inc., Canada
139	Phase II	Bemcentinib	cancer	Axl receptor tyrosine kinase inhibitor	Bergenbio, Norway
140	Phase II	Gelsolin (rhu-pGSN)	bronchitis; cystic fibrosis; systemic inflammatory response syndrome	protein replacement	Bioaegis Therapeutics Inc., USA
141	Phase II	BIO-11006	cancer	myristoylated alanine rich C kinase substrate inhibitor	Biomarck Pharmaceuticals Ltd., USA
142	Phase II	BLD-2660	fibrosis	calpain inhibitor; virus replication inhibitor	Blade Therapeutics Inc., USA; Clinipace Worldwide, USA
143	Phase II	Abatacept	juvenile rheumatoid arthritis; psoriatic arthritis; rheumatoid arthritis	T cell activation inhibitor	Bristol Myers Squibb Co., USA
144	Phase II	Ozanimod	multiple sclerosis	sphingosine 1 phosphate receptor modulator	Bristol Myers Squibb Co., USA; Celgene Corp., USA; Laval University, Canada
145	Phase II	Clevudine	hepatitis B	DNA-directed DNA polymerase inhibitor	Bukwang Pharmaceutical Co. Ltd., South Korea
146	Phase II	Desidustat	anaemia	hypoxia-inducible factor-proline dioxygenase inhibitor	Cadila Healthcare Ltd., India
147	Phase II	Pegylated Interferon - α2b	hepatitis B; hepatitis C; malignant melanoma	interferon alpha stimulant	Cadila Healthcare Ltd., India
148	Phase II	Auxora (CM-4620-IE)	pancreatitis	immunosuppressant; ORAI1 protein inhibitor; STIM1 protein inhibitor	Calcimedica Inc., USA
149	Phase II	Thalidomide	leprosy; multiple myeloma	angiogenesis inhibitor; immunosuppressant; tumour necrosis factor inhibitor	Celgene Corp., USA
150	Phase II	Mesenchymal stem cells (MSCs)	regenerative therapy for various injuries	allogeneic cell-based therapy	Celltex Therapeutics Corp., USA
151	Phase II	CERC-002	Crohn’s disease	tumour necrosis factor ligand superfamily member 14 inhibitor	Cerecor Inc., USA
152	Phase II	Clazakizumab	psoriatic arthritis; rheumatoid arthritis; renal transplant rejection	IL-6 inhibitor	Columbia University, USA; NYU Langone Health, USA; Vitaeris INC, Canada; Cedars-Sinai Medical Center, USA; Johns Hopkins University, USA; Medical University of Vienna, Austria
153	Phase II	TXA-127	duchenne muscular dystrophy; epidermolysis bullosa; limb girdle muscular dystrophies; marfan syndrome; muscular dystrophies; stroke	proto-oncogene protein c-mas-1 agonist	Constant Therapeutics Inc., USA
154	Phase II	Garadacimab (CSL-312)	hereditary angioedema	factor XIIa inhibitor	CSL Behring, USA
155	Phase II	DUR-928	acute kidney injury; alcoholic hepatitis; liver disorders	inflammation mediator modulator; lipid modulator	Durect Corp., USA
156	Phase II (IND filed)	Dantrolene (Ryanodex)	spinal cord injury; stroke; cerebral palsy; multiple sclerosis	ryanodine receptor calcium release channel modulator	Eagle Pharmaceuticals Inc., USA
157	Phase II	Peginterferon lambda	hepatitis D	interleukin 29 receptor agonist	Eiger Biopharmaceuticals Inc., USA; Stanford University School of Medicine, USA
158	Phase II	LY-3127804	tumor	angiopoietin-2 inhibitor	Eli Lilly and Co., USA
159	Phase II	M-5049	immunological disorders	toll-like receptor 7 antagonist; toll-like receptor 8 antagonist	EMD Serono Inc., USA
160	Phase II	Leukocyte cell therapy (Allocetra)	graft-versus-host disease; inflammation	cell replacement; immunomodulator	Enlivex Therapeutics Ltd., Israel; Israel Innovation Authority
161	Phase II	Itolizumab	plaque psoriasis	CD6 antigen inhibitor	Equillium Inc., USA; Biocon Ltd., India
162	Phase II	Tecarfarin	thromboembolism; thrombosis	vitamin K epoxidase inhibitor	Espero Biopharma Inc., USA
163	Phase II	Niclosamide (FW-1022)	viral infection	angiotensin type 2 receptor modulator; virus replication inhibitor	Firstwave Bio Inc., USA
164	Phase II	Quinine (GLS-1200)	sinusitis	G protein-coupled receptor agonist	Geneone Life Science Inc., South Korea
165	Phase II	Otilimab	rheumatoid arthritis	granulocyte macrophage colony stimulating factor antagonist	Glaxosmithkline, UK
166	Phase II	Antroquinonol (Hocena)	atopic dermatitis; cancer; hepatitis B; hyperlipidaemia	epidermal growth factor receptor modulator	Golden Biotechnology Corp., Taiwan
167	Phase II	GAMUNEX-C (intravenous immune globulin)	chronic inflammatory demyelinating polyradiculoneuropathy; idiopathic thrombocytopenic purpura; immunodeficiency disorders	amyloid beta-protein inhibitors; immunostimulants	Grifols, Spain; U.S. Biomedical Advanced Research and Development Authority, USA; FDA
168	Phase II	Allogeneic stem cell therapy (HLCM-051)	graft-versus-host disease	cell replacements	Healios K.K., Japan
169	Phase II	Aprepitant (Cinvanti)	chemotherapy-induced nausea and vomiting	neurokinin 1 receptor antagonists; Virus replication inhibitor	Heron Therapeutics Inc., USA
170	Phase II	HB-adMSCs	Alzheimer’s disease; rheumatoid arthritis; traumatic brain injuries	cell replacements	Hope Biosciences LLC, USA
171	Phase II	Genistein	acute radiation syndrome	antioxidant; apoptosis inhibitor; haematopoietic cell growth factor stimulant; protein tyrosine kinase inhibitor	Humanetics Corp., USA
172	Phase II	Interleukin-2	rheumatoid arthritis; lupus	regulatory T-lymphocyte stimulant	Iltoo Pharma, France; Assistance Publique - Hopitaux de Paris, France
173	Phase II	CYTO-201	immunomodulator; opioid receptor antagonist	autoimmune disorders; cancer	Immune Therapeutics Inc., USA; Cytocom Inc., USA
174	Phase II	Vidofludimus (IMU-838)	Crohn’s disease; multiple sclerosis	dihydroorotate dehydrogenase inhibitor; virus replication inhibitor	Immunic Inc., USA
175	Phase II	Xpro-1595	Alzheimer’s disease; nonalcoholic steatohepatitis; solid tumours	immunostimulant; tumour necrosis factor alpha inhibitor	Inmune Bio Inc., USA
176	Phase II	Avdoralimab	liver cancer; nonsmall cell lung cancer; solid tumours	complement C5a receptor antagonist	Innate Pharma, France; Marseille Immunopole, France
177	Phase II	Nangibotide	myocardial infarction; septic shock	TREML1 protein inhibitor	Inotrem, France
178	Phase II	Hydroxychloroquine + azithromycin	malaria; acute sinusitis; bacterial infections	autophagy inhibitor; phospholipase A2 inhibitor; 50S ribosomal subunit inhibitor	Intermountain Healthcare, USA; The Lundquist Institute, USA
179	Phase II	Tocilizumab biosimilar	rheumatoid arthritis	IL-6 receptor antagonist	Jinyu Biotechnology Co. Ltd., China
180	Phase II	Decitabine	acute myeloid leukaemia; chronic myeloid leukaemia; myelodysplastic syndromes	DNA methylation inhibitor	Johns Hopkins University, USA
181	Phase II	Crizanlizumab	vaso-occlusive crisis	P selectin inhibitor	Johns Hopkins University, USA; Novartis AG, Switzerland; Socar Research SA, Switzerland; Brigham and Women’s Hospital, USA
182	Phase II	Alvelestat (MPH-966)	alpha 1-antitrypsin deficiency; type 2 diabetes mellitus	leucocyte elastase inhibitor	Kafrelsheikh University, Egypt
183	Phase II	KB-109	bacterial infections	microbiome modulator	Kaleido Biosciences Inc., USA
184	Phase II	Selinexor (KPT-330, Xpovio)	diffuse large B cell lymphoma; multiple myeloma	exportin-1 protein inhibitor	Karyopharm Therapeutics Inc., USA
185	Phase II	Telmisartan	cardiovascular disorders; hypertension	ACE inhibitors; angiotensin type 2 receptor antagonist	Laboratorio Elea Phoenix, Argentina; University of Hawaii, Honolulu
186	Phase II	Fenretinide (LAU-7b)	cystic fibrosis	retinoic acid receptor agonist	Laurent Pharmaceuticals Inc., Canada
187	Phase II	Tranexamic acid (LB-1148)	cardiogenic shock; post-surgical adhesions; postoperative ileus; septic shock	antifibrinolytic agent; serine protease inhibitor	Leading Biosciences Inc., USA
188	Phase II	Secukinumab	ankylosing spondylitis; plaque psoriasis; psoriatic arthritis	IL17A protein inhibitor	Lomonosov Moscow State University, Russia
189	Phase II	Thiolanox	cystic fibrosis; mycobacterial infections	guanylate cyclase stimulant	Mallinckrodt plc, UK; Novoteris LLC, USA
190	Phase II	OT-101 + artemisinin	cancer; malaria	transforming growth factor beta2 inhibitor; virus replication inhibitor; free radical-mediated damage	Mateon Therapeutics Inc., USA
191	Phase II	Fisetin	aging; cancer	antioxidant; PI3K/AKT/mTOR pathway inhibitor; anti-proliferative agent; topoisomerase inhibitor; inhibitor of pro-inflammatory cytokines	Mayo Clinic, USA
192	Phase II	Ibudilast (MN-166)	asthma; stroke; multiple sclerosis	phosphodiesterase inhibitor	Medicinova Inc., USA
193	Phase II	Fingolimod (Gilenya)	multiple sclerosis	apoptosis stimulant; immunosuppressant; sphingosine 1 phosphate receptor modulator	Novartis, Switzerland
194	Phase II	NanO2	acute ischemic stroke	diagnostic imaging enhancer; oxygen carrier	Nuvox Pharma LLC, USA
195	Phase II	Camostat mesylate	pancreatitis	serine protease TMPRSS-2 inhibitor; membrane fusion inhibitor	Ono Pharmaceuticals Inc., Japan
196	Phase II	Calcifediol (Rayaldee)	secondary hyperparathyroidism	calcitriol receptor agonist	Opko Health Inc., USA
197	Phase II	OP-101	adrenoleucodystrophy	I-kappa B kinase inhibitor; NF kappa B kinase inhibitor; nuclear importation inhibitor	Orpheris Inc., USA
198	Phase II	Vafidemstat	autistic disorder; schizophrenia; Alzheimer’s disease; multiple sclerosis	lysine specific demethylase 1 inhibitor; monoamine oxidase B inhibitor	Oryzon Genomics, Spain
199	Phase II	Iloprost	arterial occlusive disorders; pulmonary arterial hypertension	epoprostenol agonist	Rigshospitalet, Denmark
200	Phase II	Tofacitinib	psoriatic arthritis; rheumatoid arthritis; ulcerative colitis	immunosuppressant; janus kinase inhibitor	Pfizer Inc., USA; Yale University, USA; Universita Politecnica delle Marche, Italy
201	Phase II	Plitidepsin (Aplidin)	multiple myeloma	apoptosis stimulant; cell cycle inhibitor; protein synthesis inhibitor	Pharmamar SA, Spain
202	Phase II	PB-1046	cardiomyopathies; pulmonary arterial hypertension	vasoactive intestinal peptide type II receptor agonist	Phasebio Pharmaceuticals Inc., USA
203	Phase II	PUL-042	chronic obstructive pulmonary disease; haematological malignancies	immunostimulant; toll-like receptor agonist	Pulmotect Inc., USA
204	Phase II	AMY-101	gingivitis; periodontitis; paroxysmal nocturnal haemoglobinuria	complement C3 inhibitor	Amyndas Pharmaceuticals Inc., USA; Quartesian LLC, USA
205	Phase II	RBT-9	kidney diseases	organ protective activity	Renibus Therapeutics Inc., USA; Cascade Chemistry Inc., USA
206	Phase II	Interleukin-7 (CYT-107)	cancer, AIDS, sepsis	IL-7 receptor agonist	Revimmune, USA; University Hospital, Limoges, France; Amarex Clinical Research, USA; Memorial Sloan Kettering Cancer Center, USA; Washington University School of Medicine, USA
207	Phase II	EIDD-2801	chikungunya, ebola, influenza	virus replication inhibitor	Ridgeback Biotherapeutics LP, USA; Emory University, USA; Merck & Co. Inc., USA
208	Phase II	Gimsilumab	ankylosing spondylitis	granulocyte macrophage colony stimulating factor antagonist	Roivant Sciences Ltd., Switzerland; Altasciences Co. Inc.
209	Phase II	STI-5656 (abivertinib maleate)	cancer	epidermal growth factor receptor antagonist	Sorrento Therapeutics Inc., USA
210	Phase II	Estradiol patch	menopausal syndrome	estrogen receptor agonist	Stony Brook University Hospital, USA
211	Phase II	Interferon-beta-1a (SNG-001)	chronic obstructive pulmonary disease; influenza	immunostimulant; interferon beta stimulant	Synairgen plc, UK
212	Phase II	Axatilimab	chronic graft versus host disease	antibody inhibitor of colony stimulating factor 1 receptor	Syndax Pharmaceuticals, USA
213	Phase II	Interferon beta-1b + clofazimine	multiple sclerosis; leprosy; tuberculosis	immunomodulator; interferon beta stimulant; adenosine triphosphatase inhibitor; P-glycoprotein inhibitor; phospholipase A2 inhibitor	The University of Hong Kong, Hong Kong
214	Phase II	Anti-PD-1 antibody	Alzheimer’s disease; cancer	amyloid beta-protein inhibitor	The University of Hong Kong, Hong Kong; Queen Mary Hospital, Hong Kong; Southeast University, China
215	Phase II	Infliximab	Crohn’s disease; ulcerative colitis; rheumatoid arthritis; ankylosing spondylitis; psoriasis; psoriatic arthritis	tumour necrosis factor alpha inhibitor	Tufts Medical Center, USA; National Institutes of Health, USA
216	Phase II	Zilucoplan	paroxysmal nocturnal haemoglobinuria; myasthenia gravis	complement C5 inhibitor	Ghent University Hospital, Belgium; UCB Pharma, Belgium
217	Phase II	Tranexamic acid	cardiogenic shock; post-surgical adhesions; postoperative ileus; septic shock	antifibrinolytic agent; serine protease inhibitor	University of Alabama at Birmingham, USA; Leading Biosciences Inc., USA; Duke University, USA; The Emmes Co. LLC, USA; Eunice Kennedy Shriver National Institute of Child Health and Human Development, USA
218	Phase II	C21	idiopathic pulmonary fibrosis	angiotensin type 2 receptor agonist	Vicore Pharma, Sweden; Orphan Reach, UK
219	Phase II	Maraviroc	AIDS	CCR5 receptor antagonist; virus internalisation inhibitor	Viiv Healthcare, USA; Hospital Clinic de Barcelona, Spain; Hospital Universitario Infanta Leonor, Spain; Rhode Island Hospital, USA
220	Phase II	Merimepodib (Vicromax)	hepatitis C; psoriasis	immunosuppressant; inosine monophosphate dehydrogenase inhibitor	Viralclear Pharmaceuticals Inc., USA
221	Phase II	Elpida (Elsulfavirine)	AIDS	nonnucleoside reverse transcriptase inhibitor	Viriom Inc., USA
222	Phase II	PH-94B	social phobia	chemoreceptor cell modulator	Vistagen Therapeutics Inc., USA
223	Phase II	Fluvoxamine	obsessive-compulsive disorders; social phobia	serotonin uptake inhibitor	Washington University, USA
224	Phase II	XAV-19	viral infection	coronavirus spike glycoprotein modulator	Xenothera SAS, France; LFB SA, France; Nantes University Hospital, France; BPIfrance
225	Phase I/II	T-COVID	viral infection	immunomodulator	Altimmune Inc., USA
226	Phase I/II	CYNK-001	multiple myeloma; acute myeloid leukaemia; glioblastoma	antibody-dependent cell cytotoxicity; natural killer cell replacement	Celularity Inc., USA; Sorrento Therapeutics Inc., USA; United Therapeutics Corp., USA
227	Phase I/II	CAStem	acute lung injury	cell replacements	Chinese Academy of Sciences, China
228	Phase I/II	NKG2D-ACE2 CAR-NK cells	pneumonia	immunomodulator	Chongqing Public Health Medical Center, China; Chongqing Sidemu Biotechnology Technology Co. Ltd., China
229	Phase I/II	Brequinar	acute myeloid leukaemia	dihydroorotate dehydrogenase inhibitor; immunosuppressant	Clear Creek Bio Inc., USA
230	Phase I/II	Meplazumab	malaria; viral infection	metalloprotease inhibitor	Jiangsu Pacific Meinuoke Biopharmaceutical Co., China; Fourth Military Medical University, China
231	Phase I/II	Lanadelumab	hereditary angioedema	plasma kallikrein inhibitors	Radboud University, Netherlands; Takeda, Japan
232	Phase I/II	RAPA-501-ALLO off-the-shelf cells	amyotrophic lateral sclerosis	autologous T cell immunotherapy	Rapa Therapeutics LLC, USA; Hackensack Meridian Health, USA
233	Phase I/II	Pentoxifylline	peripheral artery disease	phosphodiesterase inhibitor	Sadat City University, Egypt
234	Phase I/II	SBI-101	acute kidney injury	immunosuppressant; stem cell modulator	Sentien Biotechnologies Inc., USA
235	Phase I/II	Ulinastatin	pancreatitis; vascular disorders	serine protease inhibitor; trypsin inhibitor	Stanford University, USA
236	Phase I/II	Tramadol	pain	opioid mu receptor agonist; serotonin uptake inhibitor	Tanta University, Egypt
237	Phase I/II	TL-895	viral infection	tyrosine kinase inhibitor	Telios Pharma Inc., USA
238	Phase I	Agent-797	cancer; viral infection	immunologic cytotoxicity; natural killer cell replacement	Agenus Inc., USA
239	Phase I	Ampion	osteoarthritis; eye disorders	cytokine inhibitor; inflammation mediator inhibitor; IL-6 inhibitor	Ampio Pharmaceuticals Inc., USA
240	Phase I	APL-9	paroxysmal nocturnal haemoglobinuria	complement C3 inhibitor	Apellis Pharmaceuticals Inc., USA
241	Phase I	Solnatide	acute lung injury, pulmonary oedema	sodium channel agonist	Apeptico Forschung und Entwicklung GmbH, Austria
242	Phase I	T-89	chronic stable angina	improve blood circulation; boost energy metabolism; reduce blood thickness	Arbor Pharmaceuticals Inc., USA; Tasly Pharmaceutical Group Co. Ltd., China
243	Phase I	BX-U001	inflammatory bowel diseases; rheumatoid arthritis	cell replacement	Baylx Inc., USA
244	Phase I	Galidesivir	zika; ebola; marburg; yellow fever	RNA replicase inhibitor	Biocryst Pharmaceuticals Inc., USA
245	Phase I	BAT-2020	viral infection	unknown	Bio-Thera Solutions, China
246	Phase I	BRII-198	viral infection	human monoclonal antibody treatment	Brii Biosciences, China; Columbia University, USA; Tsinghua University, China; 3rd People’s Hospital of Shenzhen, China; TSB Therapeutics (Beijing) Co. Ltd., China
247	Phase I	BRII-196	viral infection	human monoclonal antibody treatment	Brii Biosciences, China; TSB Therapeutics (Beijing) Co. Ltd., China
248	Phase I	CK-0802	adult respiratory distress syndrome	T lymphocyte replacement	Cellenkos Inc., USA
249	Phase I	CT-P59	viral infection	immunostimulant	Celltrion Inc., South Korea
250	Phase I	Azvudine	AIDS	reverse transcriptase inhibitor	Chinese research sponsors, China
251	Phase I	CPI-006	cancer	5-nucleotidase inhibitor	Corvus Pharmaceuticals Inc., USA
252	Phase I (pending)	Cymerus	asthma; cancer; immunological disorders; myocardial infarction; sepsis	cell replacement	Cynata Therapeutics Ltd., Australia
253	Phase I	Trans sodium crocetinate	brain metastases; glioblastoma	oxygen compound modulator	Diffusion Pharmaceuticals Inc., USA; University of Virginia, USA
254	Phase I	Convalescent Plasma; SARS-CoV-2 specific T cells	viral infection	antibody treatment; immunotherapy	Emory University, USA; University of Southern California, USA; various Singapore hospitals, Duke-NUS Graduate Medical School, Singapore; University of California, Los Angeles, USA
255	Phase I	Bacteriotherapy	diarrhoea	bacteria replacement; microbiome modulator	Exegi Pharma LLC, USA
256	Phase I	FSD-201	inflammation; pain; fibromyalgia; irritable bowel syndrome; neurological disorders	cannabinoid receptor agonist; G-protein-coupled receptor 55 modulator; peroxisome proliferator-activated receptor alpha agonist	FSD Pharma Inc., Canada
257	Phase I	IDB-003	viral infection	monoclonal antibody-based treatment	Idbiologics Inc., USA
258	Phase I	TJM-2	rheumatoid arthritis	granulocyte macrophage colony stimulating factor antagonist	I-Mab Biopharma Co. Ltd., China
259	Phase I	JS-016	viral infection	coronavirus spike glycoprotein inhibitor	Junshi Biosciences Ltd., China; Institute of Microbiology of the Chinese Academy of Sciences, China; Eli Lilly and Co., USA
260	Phase I	Proxalutamide (GT-0918)	prostate cancer; breast cancer	androgen receptor antagonist	Kintor Pharmaceutical Ltd., China
261	Phase I	Amnioboost	osteoarthritis	processed amniotic fluid supplement	Lattice Biologics Ltd., USA
262	Phase I	FT-516	acute myeloid leukaemia; B-cell lymphoma; solid tumor	antibody-dependent cell cytotoxicity; natural killer cell replacement	Masonic Cancer Center, USA; University of Minnesota, USA
263	Phase I	MK-5475	pulmonary hypertension	reduce pulmonary blood volume	Merck Sharp & Dohme Corp., USA
264	Phase I	TAK-981	non-Hodgkin’s lymphoma; solid tumor	small ubiquitin-related modifier protein inhibitor	Millennium Pharmaceuticals Inc., USA; Takeda, Japan
265	Phase I	CD-16; N-803; BM-Allo.MSC (mesenchymal stem cells)	cancer; solid tumor	IL-15 receptor agonist; cell replacement	Nantkwest Inc., USA; Immunitybio Inc., USA
266	Phase I	NT-I7 (efineptakin alfa)	breast cancer; glioblastoma; skin cancer; solid tumour	antibody-dependent cell cytotoxicity; interleukin 7 replacement; T lymphocyte stimulant	Neoimmunetech Inc., USA
267	Phase I	Idronoxil (Veyonda)	cancer	induce tumor cell apoptosis	Noxopharm Co., Australia
268	Phase I	PL-8177	inflammatory bowel diseases; ulcerative colitis	melanocortin type 1 receptor agonist	Palatin Technologies Inc., USA
269	Phase I	LYT-100 (deupirfenidone)	lymphoedema	collagen inhibitor; cytokine inhibitor	Puretech Health plc, USA
270	Phase I	Fostamatinib (Tavalisse)	idiopathic thrombocytopenic purpura	syk kinase inhibitor	Rigel Pharmaceuticals Inc., USA
271	Phase I	SAB-185	viral infection	immunomodulator	Sab Biotherapeutics, USA; U.S. Department of Defense; CSL Behring LLC, USA
272	Phase I	SAR-443122	psoriasis; rheumatoid arthritis	RIPK1 protein inhibitor	Sanofi, France
273	Phase I	STI-1499 (Covi-Shield)	COVID-19	block viral binding to receptor	Sorrento Therapeutics Inc., USA
274	Phase I	STI-4398 (Covi-Shield)	COVID-19	ACE modulator; virus replication inhibitor	Sorrento Therapeutics Inc., USA; University of Texas Medical Branch at Galveston, USA; Mount Sinai Health System, USA
275	Phase I	TAK-671	pancreatitis	immunomodulator; trypsin inhibitor	Takeda Pharmaceutical Co. Ltd., Japan
276	Phase I	Gamma-delta T	cancer	immunotherapy	TC Biopharma Ltd., UK
277	Phase I	Novaferon	hepatitis B; neuroendocrine tumour; cancer	interferon stimulants	Zhejiang University Medical School, China
278	Phase I	TD-0903	acute lung injury	janus kinase inhibitor	Theravance Biopharma Inc., Cayman Islands
279	Phase I	TRV-027	heart failure; adult respiratory distress syndrome	angiotensin type 1 receptor antagonist; beta-arrestin stimulant	Trevena Inc., USA; Imperial College London, UK
280	Phase I	TY-027 (bifunctional peptide derivative)	viral infection	virus internalisation inhibitor	Tychan Pte Ltd., Singapore
281	Phase I	Decidual stromal cells	ARDS	reduce lung inflammation	University Health Network, Canada; Oslo University Hospital, Norway
282	Phase I	Leflunomide	psoriatic arthritis; rheumatoid arthritis	inhibit dihydroorotate dehydrogenase	University of Chicago, USA
283	Phase I	Umbilical cord-derived mesenchymal stem cells (intravenous)	graft-versus-host disease	cell replacement	Wuhan Hamilton Biotechnology Co. Ltd., China
284	Phase I	Plasma treatment	COVID-19	natural antibodies against COVID-19	Xbiotech Inc., USA; Biobridge Global, USA

For further information visit the following links: https://clinicaltrials.gov, https://www.bioworld.com/COVID19products#vac1 & https://adisinsight.springer.com.

Human monoclonal antibody-based drug sarilumab which inhibits IL-6 receptor is now being tested against COVID-19 (Lamb and Deeks, 2018). Monoclonal antibody-based rheumatoid arthritis drug tocilizumab which is also an inhibitor of IL-6 receptor found to be effective in critically ill COVID-19 patients with cytokine storms and elevated IL-6 levels (Venkiteshwaran, 2009; Chakraborty et al., 2020e; Luo et al., 2020; Saha et al., 2020b). Another monoclonal antibody-based drug leronlimab (PRO 140) known to bind to the CCR5 receptor on the CD4^+^ T lymphocytes is now being tested in COVID-19 clinical trials (Pugach et al., 2008). The proinflammatory chemokine such as C-C motif chemokine ligand 5 (CCL5) also recognized as regulated through activation, normal T cell expression, and secretion (RANTES), binds to its receptor C-C chemokine receptor type 5 (CCR5) and activates inflammatory responses by directing immune cells to the inflammation site (Vangelista and Vento, 2018). Blocking of CCR5 by leronlimab found to reduce serum IL-6 levels, which is linked with cytokine storm, in critical COVID-19 patients (Patterson et al., 2020). Interleukin-6 (IL-6) plays a vital role in inducing cytokine storm in critical COVID-19 patients and a reduction in IL-6 levels by anti-inflammatory drugs is expected to ease CRS and reduce viral loads (Zhang C. et al., 2020).

Anti-inflammatory corticosteroid drug dexamethasone has been suggested recently to treat severe COVID-19 patients with CRS. Dexamethasone reduces the production of cytokines but is also known to inhibit the protective functions of T cells and B cells. Therefore, the drug may be used selectively in some severe COVID-19 cases, but its general usage in other COVID-19 patients may cause more harm by increasing the viral load in patients due to the inhibition of protective antibody production (Lee et al., 2004; Russell et al., 2020). A recent clinical trial has shown that dexamethasone reduced the death rate among severe COVID-19 patients who needed oxygen support (**Table 2**). A recent study with severe COVID-19 patients found a direct link between C-reactive protein (CRP) and inflammation where higher CRP levels in the blood show greater inflammation. The study also showed that dexamethasone should only be used in severe COVID-19 patients with CRP levels above 20 mg per deciliter of blood, and the use of dexamethasone should be avoided in COVID-19 patients (under ventilator support) with CRP level below 10 as it may turn out to be fatal (Keller et al., 2020).

Anti-inflammatory rheumatoid arthritis drug baricitinib was found to reduce the levels of cytokines, including IFN-*γ* in severe COVID-19 patients (Huang et al., 2020). High levels of proinflammatory cytokines and chemokines including INF-*γ* in the plasma causes inflammatory cytokine storm that may lead to the occurrence of ARDS in virus-infected patients, therefore use of anti-inflammatory drugs in COVID-19 may help in the reduction of severe symptoms (Ye et al., 2020). Another rheumatoid arthritis drug anakinra is known to block the IL-1 receptor and reduce the inflammatory effects of IL-1. Survival rate within patients with hyperinflammatory conditions was found to increase when treated with anakinra (Shakoory et al., 2016).

## Convalescent Plasma

### Therapeutics

Convalescent plasma (CP) therapy is another procedure now being tested for COVID-19. This therapy is very simple yet effective, where the serum from the COVID-19 recovered persons can treat new patients (Mire et al., 2016). Recovered patients who have suffered from COVID-19 should have an elevated amount of polyclonal antibodies raised by the immune system to prevent new rounds of infection by SARS-CoV-2. Therefore, the plasma harvested from the recovered patients can be transfused to the patients who have contacted the virus (Marano et al., 2016). As the application of convalescent plasma is a well-known procedure and has been utilized before by medical practitioners, it should not be too difficult to apply this procedure to SARS-CoV-2 infected patients. Convalescent plasma has been used previously during the Ebola outbreak in 2014 and was found to be effective in treating Ebola patients (Kraft et al., 2015). A recent report has shown that CP acquired from recovered patients was effective in treating new COVID-19 infected persons (Duan et al., 2020). One problem using CP therapy is the significant variability of potency that has been found in the sera of recovered patients in neutralizing the antigen, making it a less viable option in the treatment of patients (Marano et al., 2016). Also, if the number of infected patients is much higher than the recovered patients, it would be tough to get enough CP for transfusion. Although CP therapy is being considered or used for the COVID-19 treatment, ultimately, it has limited scope in controlling the outbreak at present.

## Interferon Therapy

### Therapeutics

Type I interferons (IFN-I) stimulate the immune system upon viral infection by activating macrophages, natural killer cells, etc. and are expected to hinder SARS-CoV-2 infection (Samuel, 2001; Belhadi et al., 2020; Martinez, 2020). IFN-I is secreted by several cells when the pattern recognition receptors (PRRs) binds viral particles (Liu, 2005). IFN-I is recognized by the interferon-α/β receptor (IFNAR) in the plasma membrane. Upon binding of IFN-I, IFNAR induces the phosphorylation of several transcriptional factors, including STAT1. Once localized in the nucleus, STAT1 activates interferon-stimulated genes (ISGs), including PRRs, which further helps in decreasing membrane fluidity that inhibits viral entry through the membrane (Totura and Baric, 2012; Schneider et al., 2014). Although interferon treatment against SARS-CoV and MERS-CoV has shown variable efficiency (Stockman et al., 2006), the IFNβ subtype appears to work well in COVID-19 treatment if administered in the early stages of infection (Sallard et al., 2020). The side effects of interferon treatment could be toxic to a patient, especially when the patient is at critical stages of infection. Therefore, it is recommended to use this therapy in the early stages of infection.

## Membrane Fusion Inhibitors

### Therapeutics

Well-known antimalarial drugs chloroquine and its less toxic derivative hydroxychloroquine, both known to elevate the pH of endosomes/lysosomes that blocks membrane fusion and inhibits viral infection (Mauthe et al., 2018). Also, chloroquine found to impede glycosylation of the ACE2 receptor, which may inhibit the virus from receptor binding (Vincent et al., 2005). Both of these drugs helped inhibit this virus in the *in vitro* assays (Liu J. et al., 2020; Wang M. et al., 2020). However, some studies have raised concerns about the effectiveness of chloroquine/hydroxychloroquine in treating COVID-19 patients as these repurposed drugs were found to possess several side effects (Chary et al., 2020; Chen J. et al., 2020; Gautret et al., 2020; Kamp et al., 2020).

Current reports suggested that the influenza drug umifenovir is effective in reducing symptoms of COVID-19 (Zhang J. N. et al., 2020). Umifenovir (Arbidol) intercalates with the membrane lipids to inhibit the fusion between the virus particle and host membrane, which blocks the entrance point of the virus inside the host cell (Villalaín, 2010; Blaising et al., 2014). Another influenza drug oseltamivir, which reduces infection in the respiratory system by blocking viral neuraminidase and inhibits viral particles from escaping host cells, was found to be effective in the COVID-19 outbreak in China (Uyeki, 2018; Wang D. et al., 2020).

Coronaviruses use several modes of endocytosis (clathrin‐ or caveolin-mediated, or by the formation of lipid rafts) depending on the virus and cell type, and therefore, blocking of the endocytic pathways could be a promising strategy for the development of antiviral drugs (Glebov, 2020; Yang and Shen, 2020). Several anti-endocytotic drugs (e.g., chlorpromazine, bafilomycin, etc.) that are known to inhibit clathrin-or caveolin-mediated endocytosis proposed to have therapeutic activities against coronaviruses including SARS-CoV-2 (Yang and Shen, 2020). In lung AT2, alveolar epithelial cells, AAK1 regulates endocytosis, and baricitinib inhibits AAK1 with high affinity. Therefore, researchers argue that baricitinib could be one of the potential drugs against COVID-19 (Richardson et al., 2020). However, others argue that baricitinib also inhibits the JAK-STAT mediated signaling pathway which affects the interferon-mediated immune response. It might have a fatal effect on COVID-19 patients (Favalli et al., 2020). Clinical trials are currently underway to find out whether the drug has any positive effect in treating COVID-19 patients.

## Protease Inhibitors

### Human Protease Inhibitors (Therapeutics)

Proprotein convertases (PCs) are essential for turning precursor proteins into their active forms, e.g., furin and other proteases that control viral host cell entry and infectivity (Yamada et al., 2018; Izaguirre, 2019). Host proteases cleaved the coronavirus S proteins, including furin, TMPRSS2 (transmembrane protease serine protease 2), trypsin, cathepsin, etc., and the availability of these proteases in the infected cells are important for subsequent host cell entry (Ou et al., 2020). Furin or trypsin dependent proteolytic cleavage of the viral (SARS-CoV) S protein at two distinct sites was found to be essential for priming and subsequent membrane fusion with the host cell (Belouzard et al., 2009). MERS-CoV spike protein was also found to be activated by furin cleavage (Millet and Whittaker, 2014). Similarly, the S protein of SARS-CoV-2 has a putative cleavage site (furin) between S1 and S2 subunits, but whether it is cleaved during the priming event remains elusive (Ou et al., 2020). Another serine protease TMPRSS2 was found to be crucial for S protein priming in both SARS-CoV-2 and SARS-CoV (Matsuyama et al., 2010; Shulla et al., 2011; Iwata-Yoshikawa et al., 2019; Hoffmann et al., 2020). For SARS-CoV, it is the availability of specific proteases that appears to be the determinant factor to choose whether it enters the host cell *via* the cell surface or by using the endosomal cathepsin L-mediated pathway for viral entry. So, non-appearance of the host proteases within the cell surface, SARS-CoV invade host cells though a pathway (endosomal pathway) where cathepsin L activates the spike protein, allowing the association of the viras particle and endosome membranes (Simmons et al., 2004; Kam et al., 2009; Chan et al., 2013).

Previous studies have shown that the dual treatment of an inhibitor of TMPRSS2- camostat mesylate and an inhibitor of cathepsin L efficiently blocked host cell entry of SARS-CoV. This competent inhibition could be attributed to the double barrier of entry for SARS-CoV from the surface of a cell and through the endosomal pathway (Kawase et al., 2012). Serine protease inhibitor camostat mesylate was found to block TMPRSS2-mediated priming of spike protein and inhibits COVID-19 infection in lung cells *in vitro* (Hoffmann et al., 2020). Another TMPRSS2 inhibitor drug nafamostat mesylate was found to inhibit the membrane fusion of MERS-CoV and expected to have similar effects on this virus (Yamamoto et al., 2016; Hoffmann et al., 2020). These observations suggest that this protease inhibitor, camostat mesylate, and a cathepsin inhibitor can be used as antiviral drugs to prevent cathepsin L and TMPRSS2 -mediated SARS-CoV-2 infection.

One problem with using human protease inhibitors as antiviral drugs is that they might affect the normal physiological processes in the human cells, which may lead to further complications or side effects. Therefore, human protease inhibitors may be used in combinatorial therapies with other antiviral drugs which would allow using a less concentration of protease inhibitors to minimize side effects while keeping stronger efficacy. However, no human protease inhibitor has been approved as of now to use in treating viral infections despite having several experimental reports on their effectiveness as antiviral drugs (Steinmetzer and Hardes, 2018).

### Viral Protease Inhibitors (Therapeutics)

In coronavirus, chymotrypsin-like protease (3CL^pro^ or M^pro^) is the main protease, and along with papain-like protease (PL^pro^) it processes the polyproteins pp1ab and pp1a (Brierley et al., 1989; Gorbalenya et al., 2006). These two proteases are attractive targets for designing drugs to inhibit cleavage functions and render the virus non-functional (Anand et al., 2003; Yang et al., 2003; Ratia et al., 2008; Hilgenfeld, 2014; Arya et al., 2020; Wu C. et al., 2020). The structures of M^pro^ from SARS-CoV-2 and SARS-CoV are known. Hence, the designing of drugs to inhibit the protease has been accelerated (Xue et al., 2008; Zhang L. et al., 2020). An α-ketoamide inhibitor has been identified that blocks SARS-CoV-2 M^pro^ from performing its functions shown in mice (Zhang L. et al., 2020). HIV protease inhibitor drug lopinavir/ritonavir was found to be useful in decreasing viral loads in COVID-19 patients (Lim et al., 2020). However, in clinical trials on COVID-19 patients, the HIV drug was found to be ineffective (Cao et al., 2020). Another HIV protease inhibitor darunavir is also under clinical trials to find out its efficacy in treating COVID-19 (Santos et al., 2019). *In vitro* studies have shown that several other antiretroviral protease inhibitors (e.g., nelfinavir, etc.) were highly effective in inhibiting coronaviruses (Yamamoto et al., 2004). However, the failure of Kaletra (lopinavir/ritonavir) has shown that protease inhibitors optimized for HIV are unlikely to be effective against SARS-CoV-2 as the proteases expressed by these two viruses are structurally different. Nonetheless, some efficacy against SARS-CoV-2 has been shown by HIV protease inhibitors under *in vitro* conditions and some of these inhibitors are also under various clinical trials to confirm their effectiveness against COVID-19 (**Table 2**). However, protease inhibitors specific for HIV protease (e.g., darunavir, etc.) are doubtful to be effective against SARS-CoV-2 protease because of the structural dissimilarities between them.

## Replicase Inhibitors

### Therapeutics

Another attractive target for drug development is the SARS-CoV-2 RNA-dependent RNA polymerase (RdRp), as this is the main molecule for the replication/transcription complex in coronaviruses. The cryo-EM structure of SARS-CoV-2 RdRp (nsp12) has been elucidated recently, along with cofactors nsp7 and nsp8 (Gao Y. et al., 2020). The structure derived using cryo-EM methodology also explained how the drug remdesivir binds to the RdRp (Gao Y. et al., 2020). The nucleotide analog remdesivir has been shown to inhibit RdRp in SARS-CoV (Agostini et al., 2018; Saha et al., 2020a), MERS-CoV (Gordon et al., 2020), and SARS-CoV-2 (Holshue et al., 2020; Wang M. et al., 2020). In a recent study, remdesivir was found to provide benefit to the majority of COVID-19 patients who needed oxygen support (Grein et al., 2020). European Medicines Agency (EMA) has given conditional marketing approval to Veklury (remdesivir) for the therapy of critical COVID-19 patients (12 years of age or higher) with pneumonia and under oxygen support. Remdesivir is the first drug to get the required authorization to use in the EU for the treatment of COVID-19 (**Table 2**).

Other nucleotide/nucleoside analogs, e.g., sofosbuvir (Gane et al., 2013; Appleby et al., 2015; Ju et al., 2020), and ribavirin (Elfiky, 2020), were also found to be effective in inhibiting RdRp. Favipiravir, which has a structural similarity with nucleoside analogs, found to be effective in COVID-19 clinical trials (Chen C. et al., 2020). Another nucleoside analog galidesivir (BCX-4430) was found effective in several infectious diseases, including Ebola, Zika, etc., and maybe useful in COVID-19, too (Taylor et al., 2016; Eyer et al., 2019). Similar antiviral drugs, triphosphate forms of AZT (3’-azido-3’-deoxythymidine triphosphate), and alovudine (3’-fluoro-3’-deoxythymidine triphosphate) were also predicted to inhibit SARS-CoV-2 RdRp (Ju et al., 2020). The guanosine analog ribavirin not only inhibits viral RdRp by directly interfering with it but also interferes with the RNA capping by inhibiting inosine monophosphate dehydrogenase enzyme to impede guanosine production in the host cell (Graci and Cameron, 2006; Khalili et al., 2020). Interestingly, antiparasitic drug ivermectin was found to suppress SARS-CoV-2 replication in cell culture efficiently (Caly et al., 2020). Ivermectin was predicted to inhibit the maturation of viral proteins by blocking IMPα/β1-mediated nuclear import (Wagstaff et al., 2012; Yang et al., 2020).

## Nucleic Acid–Based Solutions

### Vaccines

The advantages of nucleic acid-based vaccines are that they can be quickly constructed and can induce strong cell-mediated and humoral immune responses even in the absence of an adjuvant (Du et al., 2009). During the Zika virus outbreak, DNA vaccines were the first to enter clinical trials (Prompetchara et al., 2020). A DNA vaccine is a new and innovative mode of vaccination involved in direct injection of a plasmid encoding the antigens (Shang et al., 2020). Certain advancements like the use of electroporation for delivering the plasmid and use of adjuvant further increases the efficacy by invoking better immune response. Several organizations are working for pre-clinical trials of DNA vaccines against COVID-19 (Liu, 2019) (**Table 1**). DNA vaccines against COVID-19 mainly encode different forms of the SARS-CoV-2 S protein that was found to stimulate both cellular and humoral immune responses in mice, guinea pigs, and rhesus macaques (Amanat and Krammer, 2020; Smith et al., 2020; Yu et al., 2020). However, there is a risk of integration and mutation of DNA vaccines within the host genome. Being safer, mRNA vaccines stand as a promising alternative to DNA and other conventional vaccine approaches because of its safety and quick development (Liu, 2019). So far, several organizations are working on developing an mRNA-based vaccine for SARS-CoV-2. Small interfering RNA (siRNA) based vaccines are also being developed targeting conserved regions on the SARS-CoV-2 genome, especially 3CL^pro^, RdRp, and spike protein, to degrade viral mRNAs resulting inhibition of translation (Liu C. et al., 2020).

### Therapeutics

Double-stranded RNA drug rintatolimod is now being tested for COVID-19, which stimulates the innate immune system by binding to one of the PRRs named TLR-3 found in the endosomal membrane. Once rintatolimod binds to TLR-3, the host cell gets a signal to produce interferons, which lead to various protective systems against pathogenic viruses or bacteria. Rintatolimod predicted to stimulate RNase L enzyme production, which degrades pathogenic RNAs of viruses (Gowen et al., 2007; Pardi et al., 2018).

## Conclusions

There are several new vaccines and novel therapeutic molecules which are currently under development against COVID-19 (**Tables 1** and **2**). The finding of a safe and attractive target for vaccine development is of utmost importance at this point to prevent further spread of this virus. Unfortunately, the way SARS-CoV-2 is spreading around the world and infected cases increasing exponentially, we may have to witness much bigger devastation before a cure is found. Several promising drug targets have been identified, and several organizations are working relentlessly to develop vaccines against these targets (**Table 1**). Different available antiviral drugs (repurposed) are being tested for COVID-19 in large clinical trials, as they have shown some positive effects in initial phases (**Table 2**; **Figure 1**). Contradictory reports are also started to pouring in against some antiviral therapies targeted at COVID-19, where although initial reports suggested positive effects, later others showed no effect. For example, hydroxychloroquine treatment, along with azithromycin, has shown a significant reduction of viral load in COVID-19 infected patients (Gautret et al., 2020), but subsequent report refutes that claim and showed no benefit in severe COVID-19 patients by this treatment (Molina et al., 2020). Repurposing existing antiviral drugs against COVID-19 has shown some positive effects, but further scientific results are necessary to prove whether these affect COVID-19 treatment, or we are just looking at the placebo effect which can be dangerous for patients.

Recently, some unproven theories are spreading like wildfires, which may also hinder the actual progress on the vaccine development against COVID-19. One example is the use of the BCG vaccine, which is being advocated as a potential cure for COVID-19. Countries, where people have taken the anti-tuberculosis Bacillus Calmette-Guerin (BCG) vaccine, appear to be immune from COVID-19 compared to countries where BCG vaccination is not a norm, as per some recent non-peer-reviewed reports (Hegarty et al., 2020; Miller et al., 2020). Research organizations have already started clinical trials to test the efficacy of the BCG vaccine in COVID-19. It is not clear at this point how and whether BCG vaccination helps in preventing COVID-19 at all; therefore, further research is necessary to find the link between these two.

Several vaccine clinical and pre-clinical trials are currently ongoing (**Table 1**), and even if some trials finally become successful, a preventive vaccine may not be widely available for at least another 12–18 months. For a vaccine to be successful, much time is needed to conduct proper clinical trials, especially phase III and phase IV trials where the control group is large enough to get a conclusive report (Green, 2020). Therefore, fast-tracking of any clinical trial could be potentially dangerous, and comprehensive safety tests are necessary before a vaccine can be marketed. It applies the same to any repurposed drugs that show positive effects in the initial phases of clinical trials. The catastrophic failure of the respiratory syncytial virus (RSV) vaccine in 1966 showed the importance of a proper clinical trial and advocating for fast-tracking any SARS-CoV-2 clinical trials should be avoided at this stage. The RSV vaccine failed due to the lack of antibody affinity maturation, the possibility of which should be thoroughly checked to avoid a similar situation in COVID-19 (Glezen et al., 1986).

Due to the high genome mutation rates in RNA viruses as the viral RNA polymerase (e.g., influenza virus) or reverse transcriptase (e.g., HIV) lacks proofreading activity, and therefore, it is difficult to make an effective vaccine against RNA viruses (Boutwell et al., 2010; Sanjuán et al., 2010). Although the excessive mutation rate in RNA viruses helps them to adapt quickly to the variable environmental conditions, it also makes them vulnerable because of the accumulation of lethal mutations in the essential genes. Interestingly, in SARS-CoV, the nsp14 protein found to contain an exoribonuclease domain (ExoN) that provides proofreading activity and the deletion of the gene results in a reduction of virulence (Hofer, 2013; Pachetti et al., 2020). This information is important as SARS-CoV-2 also contains a similar gene on its genome, and any proofreading activity would ensure low mutational rates during the synthesis of the viral genome, which would be helpful to design and to develop a vaccine candidate against the SARS-CoV-2 virus.

Coronaviruses are known for a long time and an extensive amount of knowledge has been gathered on SARS-CoV, despite that we still do not have a vaccine against it. We still do not have an effective vaccine against HIV or malaria, for example, although these pathogens are known to us for a long time (Boutwell et al., 2010; Rts, 2015; King, 2019). Challenges posed by these pathogens are far more complex and require an extensive investigation that may take several years to complete. Therefore, extensive safety trials in humans with sizable groups of people are needed even if data from the initial phases are encouraging. Any rush at these stages may be catastrophic if upon vaccination to people who never exposed to the virus develop serious side effects.

Reports from the recent clinical trials of two COVID-19 vaccine candidates have shown promise as they were found to be safe for human use and also induced strong immune response against SARS-CoV-2 (Beyrer et al., 2012; Zhu et al., 2020). The vaccine AZD1222 (ChAdOx1 nCoV-19) developed jointly by Oxford University and AstraZeneca provides double protection against COVID-19 by producing both antibodies and T-cells that directly kill infected cells (Beyrer et al., 2012). Another vaccine (Ad5-nCOV) developed by CanSino Biologics, China, also shown to provide protection against SARS-CoV-2 (Zhu et al., 2020). These reports instill faith that a protective vaccine would be available soon to ease the suffering that the world is facing today because of COVID-19.

The virus has locked up several parts of the world from social and economic activities, and we have no other option but to wait for the development of a vaccine against COVID-19. This situation was envisaged by several scientists earlier, but no one thought we have to witness this disaster in our lifetime. Humanity always prevailed under challenging conditions and the way many research organizations are trying to find a cure one can only hope that we could get a vaccine against COVID-19 sooner than later, but until then social distancing, rigorous testing, and isolation of infected persons in COVID-19 appears to be a potent strategy to contain the spread of the virus.

## Author Contributions

Writing—original draft: RS and ARS. Writing—review and editing: MKS, SS, SB, SM, and MB. Revising and supervising and funding acquisition: CC, ARS, and SSL.

## Funding

This research was supported by Hallym University Research Fund and by Basic Science Research Program through the National Research Foundation of Korea (NRF) funded by the Ministry of Education (NRF-2017R1A2B4012944 & NRF-2020R1C1C1008694).

## Conflict of Interest

The authors declare that the research was conducted in the absence of any commercial or financial relationships that could be construed as a potential conflict of interest.

## Abbreviations

HCoV, Human Coronavirus; HIV, Human Immunodeficiency Virus; SARS, Severe Acute Respiratory Syndrome; MERS, Middle East Respiratory Syndrome; WHO, World Health Organization; RAS, Renin-Angiotensin System; TLR, Toll-like receptors; STAT, Signal Transducer and Activator of Transcription, ACE2, Angiotensin-converting enzyme 2; AAK1, Adaptor-associated protein kinase 1; JAK-STAT, Janus kinases (JAKs), signal transducer and activator of transcription proteins; ADCC, Antibody-dependent cellular cytotoxicity; RdRp, RNA-dependent RNA polymerase; RSV, Respiratory syncytial virus; PLpro, papain-like protease; PRRs, pattern recognition receptors; ADE, antibody-dependent enhancement; ARDS, Acute Respiratory Distress Syndrome; CCL5, C-C motif Chemokine Ligand 5; BCG, Bacillus Calmette–Guérin; RBD, Receptor-binding domain.
